# Brain proteomic analysis implicates actin filament processes and injury response in resilience to Alzheimer’s disease

**DOI:** 10.1038/s41467-023-38376-x

**Published:** 2023-05-12

**Authors:** Zhi Huang, Gennifer E. Merrihew, Eric B. Larson, Jea Park, Deanna Plubell, Edward J. Fox, Kathleen S. Montine, Caitlin S. Latimer, C. Dirk Keene, James Y. Zou, Michael J. MacCoss, Thomas J. Montine

**Affiliations:** 1 Department of Biomedical Data Science, Stanford University School of Medicine, Stanford, CA 94305 USA; 2 Department of Pathology, Stanford University School of Medicine, Stanford, CA 94305 USA; 3 Department of Genome Sciences, University of Washington, Seattle, WA 98195 USA; 4 Department of Medicine, University of Washington, Seattle, WA 98195 USA; 5 Department of Laboratory Medicine and Pathology, University of Washington, Seattle, WA 98195 USA

**Keywords:** Alzheimer's disease, Alzheimer's disease, Proteomic analysis

## Abstract

Resilience to Alzheimer’s disease is an uncommon combination of high disease burden without dementia that offers valuable insights into limiting clinical impact. Here we assessed 43 research participants meeting stringent criteria, 11 healthy controls, 12 resilience to Alzheimer’s disease and 20 Alzheimer’s disease with dementia and analyzed matched isocortical regions, hippocampus, and caudate nucleus by mass spectrometry-based proteomics. Of 7115 differentially expressed soluble proteins, lower isocortical and hippocampal soluble Aβ levels is a significant feature of resilience when compared to healthy control and Alzheimer’s disease dementia groups. Protein co-expression analysis reveals 181 densely-interacting proteins significantly associated with resilience that were enriched for actin filament-based processes, cellular detoxification, and wound healing in isocortex and hippocampus, further supported by four validation cohorts. Our results suggest that lowering soluble Aβ concentration may suppress severe cognitive impairment along the Alzheimer’s disease continuum. The molecular basis of resilience likely holds important therapeutic insights.

## Abstract (web summary)

Resilience to Alzheimer’s disease (RAD) is an uncommon combination of high disease burden without dementia. The authors perform proteomic analysis of RAD brains and show lower isocortical and hippocampal soluble Aβ levels, actin filament-based processes, cellular detoxification, and wound healing are significant features.

## Introduction

Dementia in older individuals is a major medical challenge that looms as a public health disaster unless effective interventions are discovered and deployed^1^. Dementia in older individuals is a syndrome that derives from five different, prevalent diseases. While each of these diseases on its own can cause dementia, in the majority of affected individuals these diseases variably combine in a now widely validated idiosyncratic conspiracy of Alzheimer’s disease (AD), vascular brain injury (VBI), Lewy body disease (LBD), hippocampal sclerosis (HS), and limbic-associated TDP-43 encephalopathy (LATE)^2^. The resulting individually varying comorbidity confounds clinical research because of limited tools to detect each of these five diseases during life; hence, the major focus on developing biomarkers and the continued reliance on brain autopsy to evaluate comprehensively the burden of comorbidities in an individual.

Each of the five commonly comorbid diseases that can contribute to dementia in older individuals has a latent phase. The majority of people harbor a low burden of latent disease that is insufficient to cause dementia, referred to as preclinical^2^. In contrast, a minority harbor a high burden of latent disease(s) sufficient to cause dementia in others; this intriguing group is called resilient, meaning resilient to the clinical expression of dementia despite a sufficiently high burden of disease(s) (https://reserveandresilience.com/framework). Previous proteomic studies have focused on asymptomatic AD (AsymAD), which is a mixture of both preclinical and resilient cases^3–6^. Here, we present our proteomic study focusing on resilience to AD (RAD).

Latent disease confounds accurate assignment to the control group because without comprehensive neuropathologic assessment the control group will harbor unknown levels of comorbid disease(s)^2^. Comorbidity confounds accurate assignment as RAD; indeed, we have shown in multiple, large population- and community-based cohorts that the major driver of apparent RAD is not related to AD but rather undetected comorbidities that are infrequent in the cognitively resilient group but that are significantly more prevalent in dementia group^7,8^. Here, we have used comprehensive neuropathologic evaluation combined with clinical assessment proximate to death to resolve these confounders and allow accurate clinico-pathologic assignment of both controls free of clinically significant brain diseases and individuals with actual RAD^7,8^.

Most proteomic studies have evaluated only one or two isocortical regions that undergo neurodegeneration in AD without including a brain region that does not degenerate to control for coincident events that accompany dementia, like reduced activity and weight loss, that impact the brain but that are thought to be consequences rather than causes of neurodegeneration. Here, we have used data independent acquisition (DIA) MS/MS proteomics of soluble protein extracts from multiple brain regions donated by comprehensively evaluated research participants who were healthy controls (HC) free of clinically significant brain diseases, had actual RAD, or had AD dementia (ADD) without significant comorbidities^9^. Our differential expression analysis performed for each brain region identified 33 RAD-associated differentially expressed proteins (DEPs). Protein co-expression analysis revealed a group of 181 densely-interacting proteins that were significantly associated with RAD and enriched for actin filament-based process, cellular detoxification, and wound healing in isocortex and hippocampus. We further supported our findings using data from 689 human isocortical samples from four independent external cohorts that were the closest approximations of our clinico-pathologic groups. The molecular basis of RAD, a widely replicated state in older adults for which there is no experimental model, likely holds important insights into therapeutic interventions for AD.

## Results

**Fig. 1:**
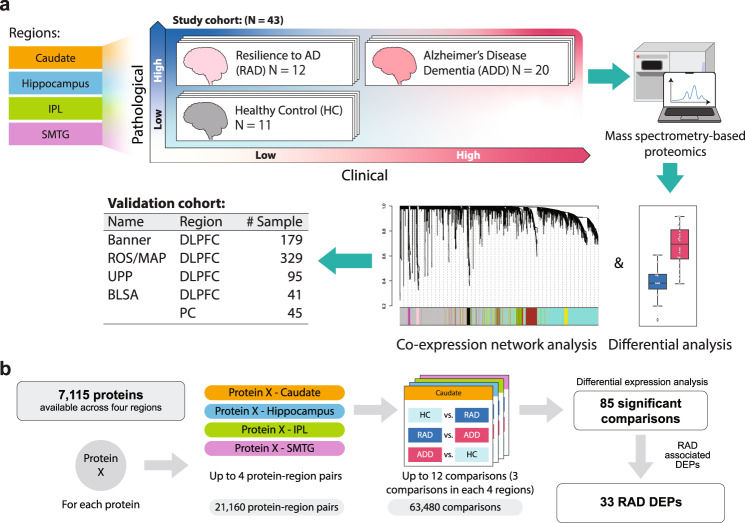
Workflow of this study. **a** Samples (*N* = 155) from up to four matched brain regions were donated by 43 research participants who were assigned to three clinico-pathologic groups: HC (healthy control), RAD (cognitive resilience to Alzheimer’s disease), or AD dementia (ADD). Samples were quantified by data independent tandem mass spectrometry and data analyzed by differential expression and co-expression network analyses. Results were compared to four independent data sets that most closely approximated our study design. **b** Illustration of differential expression analysis and summary of the final number of RAD-associated differentially expressed proteins (RAD DEPs). DLPFC dorsolateral prefrontal cortex, PC precuneus. Source data are provided as a Source Data file.

Our workflow included four different brain regions (caudate nucleus or CAUD, *N* = 38; hippocampus or HIPP, *N* = 41; inferior parietal lobule or IPL, *N* = 38; and superior and middle temporal gyrus or SMTG, *N* = 38) that were derived from 43 donors, out of 737 brain donations, who met rigorous eligibility criteria for three clinico-pathologic groups: HC (*N* = 11), RAD (*N* = 12), and ADD (*N* = 20) (Fig. 1a, Table S1, and Fig. S1). It is important to note that clinically significant co-morbidities were excluded from all groups, HC did not meet consensus criteria for AD, and RAD and ADD were matched for level of AD neuropathologic change (ADNC, *P* = 0.19). Sample preparation and DIA-based proteomics were performed exactly as described^9,10^. Briefly, in contrast to the more commonly used data-dependent acquisition method that semi-randomly selects and analyzes precursor ions for tandem mass spectrometry in real-time, DIA isolates and fragments a *m*/*z* range in a systematic and reproducible manner. Protein level results were analyzed by differential expression analysis and co-expression network analysis. Throughout, we compared our results with four independent proteomic datasets whose samples were the closest approximation of our focused study of RAD, including ROS/MAP^11^, Banner^12^, UPP^13^, and BLSA^14^ datasets (detailed cohort information is in the Methods section). A total of 7115 proteins were quantified among the 155 samples (Fig. 1b). Corrected Student’s *t*-test identified 85 significantly differentially expressed proteins (DEPs) among the three clinico-pathologic groups in at least one of the four brain regions, including 33 unique RAD-associated DEPs (RAD DEPs).

### Differential expression analysis

**Fig. 2:**
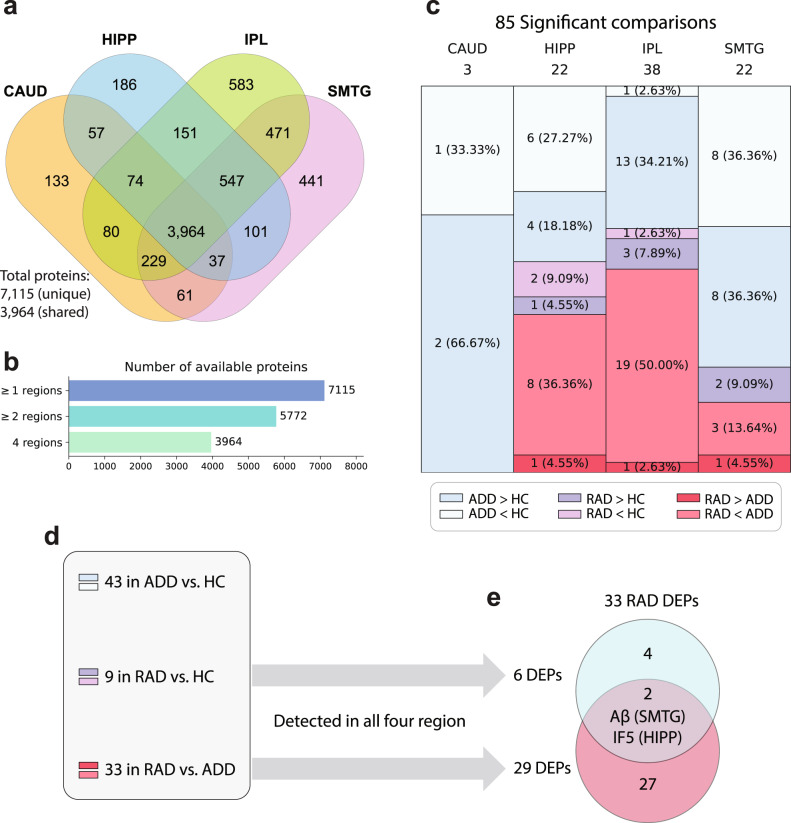
The process of deriving differentially expressed proteins (DEPs). **a** Venn diagram shows overlap in proteins quantified across four brain regions. **b** Summary of detected proteins in multiple brain regions. **c** 85 proteins were differentially expressed (FDR cut-off = 0.05) among the three clinico-pathologic groups across the four brain regions. **d** Of the 85 DEPs, 43 were significantly different between ADD versus HC, and 42 were RAD-associated, meaning significantly different between RAD and either HC or ADD in one or more regions. **e** 9 proteins were differentially expressed in RAD versus HC, and 33 proteins were differentially expressed in RAD versus ADD. Aβ and IF5 were differentially expressed in both RAD versus HC and RAD versus ADD (overlapping region in figure). We excluded RAD DEPs that were not detected in all regions, yielding 33 unique RAD DEPs measured in all four regions. CAUD caudate, HIPP hippocampus, IPL inferior parietal lobule, SMTG superior and middle temporal gyrus, HC healthy control, RAD resilience to AD, ADD Alzheimer’s disease and dementia. Source data are provided as a Source Data file.

7115 total proteins were quantified across the four brain regions (Fig. 2a), of which 5772 were detected in two or more regions and most proteins (*N* = 3964) were detected in all four regions (Fig. 2b). Total proteins first were analyzed by corrected (Benjamini–Hochberg method, FDR cutoff = 0.05) two-sided *t*-tests, yielding 85 significant protein comparisons between at least one pair of the three groups in at least one brain region (Fig. 2c). After excluding those DEPs that were not detected in all four regions, we identified 33 unique RAD DEPs (see Methods) (Fig. 2d, e), of which Aβ and IF5 were differentially expressed in both RAD versus HC and RAD versus ADD (overlapping region in Fig. 2e).

**Fig. 3:**
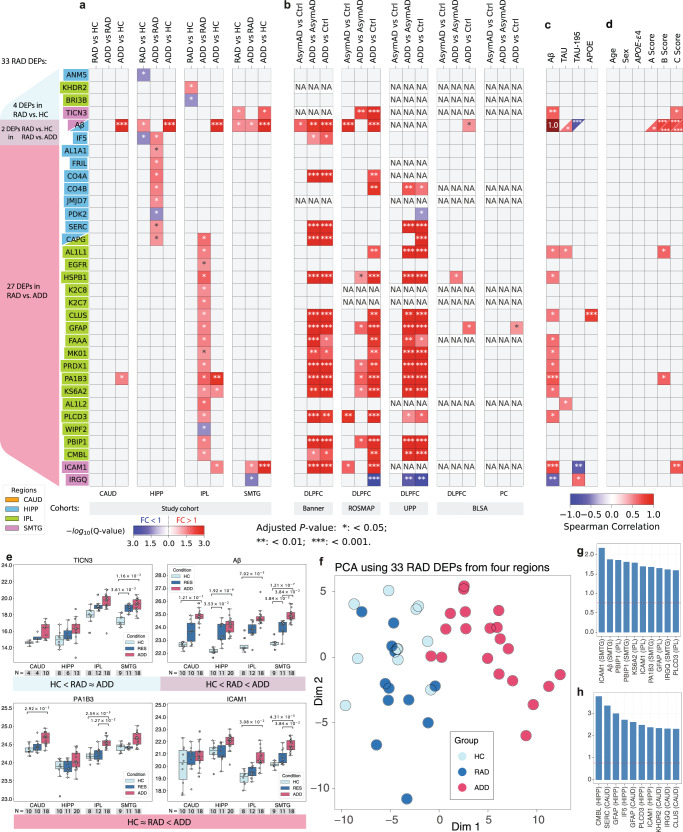
RAD DEPs among four brain regions and four external validation datasets. Results of corrected multiple comparisons among four brain regions in the study set (**a**), and the four external validation sets of which only BLSA examined two brain isocortical regions (**b**). Each column for the study set consists of three comparisons: RAD vs. HC, ADD vs. RAD, and ADD vs. HC. Two-sided Student’s *t*-test was used with the Benjamin–Hochberg procedure (FDR = 0.05) to adjust the P-values. For external datasets, each column consists of three comparisons: AsymAD vs. Ctrl, ADD vs. AsymAD, and ADD vs. Ctrl (non-significant comparisons were colored in gray; insufficient data are white and annotated with NA; fold change (FC) < 1 was colored in blue, FC > 1 was colored in red; and colors are the same for -log_10_(Adjusted *P*-value) ≥ 3). Annotations: * Adjusted *P*-value < 0.05; ** Adjusted *P*-value < 0.01, *** Adjusted *P*-value < 0.001. Correlation of expression of each RAD DEP with hallmark AD protein expression in the same brain region (**c**) and with clinical, genetic, or pathologic features of the individual (**d**). Spearman correlation test with two-sided *P*-values was used with the Benjamin-Hochberg procedure (FDR = 0.05) to adjust the *P*-values. Note: Aβ was a RAD DEP in both HIPP and SMTG, and CAPG was a RAD DEP in both HIPP and IPL. **e** Boxplots of selected RAD DEP expression. Two-sided Student’s *t*-test was used to derive *P*-values, followed with the Benjamin–Hochberg procedure (FDR = 0.05). Number of samples within each region and group are displayed under x-axes. For the boxplots, the interior horizontal line represents the median value, the upper and lower box edges represent the 75th and 25th percentile, and the upper and lower bars represent the 90th and 10th percentiles, respectively. **f** Principal component analysis (PCA) for 33 RAD DEPs in all 4 regions of each brain (original dimension: 33 × 4 = 132) colored by clinico-pathologic groups, visualized in principal dimensions 1 and 2. Variable contributions to the principal dimension 1 (**g**) and principal dimension 2 (**h**) with dashed lines in red showing variable contributions and their expected average. CAUD caudate, HIPP hippocampus, IPL inferior parietal lobule, SMTG superior and middle temporal gyrus, HC healthy control, RAD resilience to AD, ADD Alzheimer’s disease and dementia, DLPFC dorsolateral prefrontal cortex, PC precuneus. Source data of exact *P*-values are provided as a Source Data file.

We further constructed a linear model considering age, sex (female = 0, male = 1), A score (0–3), B score (0–3), and C score (0–3) as control variables. For the 29 DEPs differentially expressed in RAD vs. ADD, 22 were validated by linear model analysis (Fig. S23). Due to strong correlations between several DEPs and A, B, or C scores (for example, Aβ in Fig. 3d), several DEPs may be concealed in the linear model analysis.

### Regional analysis and validation of RAD DEPs

RAD DEPs were unevenly distributed across brain regions with the most in IPL, then HIPP, and then SMTG (Fig. 3a). Notably, RAD DEPs were largely non-overlapping across regions with only two RAD DEPs significantly different in more than one region: soluble Aβ in HIPP and SMTG, and CAPG in IPL and SMTG. All RAD DEPs were compared against four independent datasets for validation (Fig. 3b): Banner Sun Health Research Institute (Banner)^12^, Religious Orders Study and Rush Memory and Aging Project (ROS/MAP)^11^, UPenn Proteomics study (UPP)^13^, and Baltimore Longitudinal Study of Aging (BLSA)^14^. We followed the less stringent case assignment criteria used by others because of limited availability of pathologic data for the external studies and to align with previous publications; the Control (Ctrl), Asymptomatic AD (AsymAD), and ADD groups defined by others were the closest approximation of our more stringently defined groups (Table S2), and therefore should provide some level of validation^3,4^. Banner, ROS/MAP, and UPP collected proteomics data in dorsolateral prefrontal cortex (DLPFC), while BLSA collected proteomic data from both DLPFC and precuneus (PC). There was broad agreement among Banner, ROS/MAP, and UPP data; however, BLSA data did not compare well with the other three validation sets, perhaps due to the relatively limited number of samples. Despite these differences in clinico-pathologic criteria and variability in external dataset quality, 70% (23/33) of RAD DEPs in the study set were validated as AsymAD DEPs in at least one external dataset. When limiting the comparison to only isocortical regions in the study set (external dataset exclusively used isocortical regions), 71% of the 24 isocortical RAD DEPs were validated as AsymAD DEPs in one and 58% were validated in two or three external datasets.

Regional expression levels were correlated between each RAD DEP and each of four proteins thought to be central to either the etiology or pathogenesis of AD: Aβ, APOE, and TAU as well as the TAU-195 peptide (SGYSSPGSPGTPGSR) that is depleted as TAU is increasingly phosphorylated^15^ (Fig. 3c). Excluding Aβ itself, expression of 12 RAD DEPs was significantly correlated with soluble Aβ concentration; the strongest of these were PA1B3 (*P* < 0.001, for simplicity all *P* stand for adjusted *P*-value) in IPL as well as TICN3 (*P* < 0.01), ICAM1 (*P* < 0.001), and IRGQ (*P* < 0.01) in SMTG; all were positively correlated with Aβ except IRGQ levels in SMTG that were negatively correlated with Aβ levels. Expression of three proteins (Aβ in HIPP, AL1L1 in IPL, and AL1L2 in IPL) were weakly positively correlated with TAU levels in the corresponding region (*P* < 0.05 for each). Aβ (*P* < 0.001) and ICAM1 (*P* < 0.01) levels were negatively correlated with TAU-195 peptide levels in SMTG, indicating that their tissue concentrations increased with increasing tau hyperphosphorylation in this region. IRGQ levels in SMTG were positively correlated (*P* < 0.05) with TAU-195 peptide, suggesting that of all of the RAD DEPs only IRGQ levels in SMTG decreased as both Aβ and hyperphosphorylated tau increased. Only CLUS, also known as apolipoprotein J, in IPL had a significant correlation with APOE protein levels (*P* < 0.001).

Tissue levels of the RAD DEPs were then correlated with individual-level data from each of the 43 donors, and so likely limited to very strong associations. No RAD DEP’s expression correlated significantly with age, sex, or presence of *APOE* ε4 allele (Fig. 3d). Histopathologic rankings of neurofibrillary degeneration (B score)^16^ and neuritic plaque density (C score)^17^ were positively correlated with most RAD DEPs that expressed higher in ADD than HC group (Fig. S7). Aβ levels in HIPP and SMTG were strongly positively correlated with both rankings of neurofibrillary degeneration and neuritic plaque density (*P* < 0.001), aligning well with the Spearman correlations above and confirming our pathological assessments. AL1L1 and PA1B3, both in IPL, were positively correlated with ranking of neurofibrillary degeneration (*P* < 0.05). TICN3 (*P* < 0.05) and ICAM1 (*P* < 0.01) levels in SMTG were positively correlated with ranking of neuritic plaque density. Together, expression levels of five RAD DEPs were significantly correlated with both AD-related protein expression and histopathologic rankings (Aβ in SMTG and HIPP, ICAM1 in SMTG, AL1L1 in IPL, TICN3 in SMTG, and PA1B3 in IPL; Fig. 3d), while expression of IRGQ in SMTG correlated only with pathologic protein expression.

Finally, we performed principal component analysis (PCA) to inspect higher-level proteomic characteristics by summarizing protein expression levels of the 33 RAD DEPs for all 4 regions into a single value for each individual’s brain (Fig. 3e). There was broad overlap between HC and RAD groups despite one being free of clinically significant disease and the other having extensive AD neuropathologic change. Furthermore, there was near complete separation of RAD from ADD groups despite both having an equivalent high burden of AD neuropathologic change but only one succumbing to dementia. The top three contributors to PC1 (24.5% of variance) were ICAM1 (SMTG), Aβ (SMTG), and PBIP1 (IPL) (Figs. 3g and S3), while the top three contributors to PC2 (9.0% of variance) were CMBL (HIPP), SERC (CAUD), GFAP (HIPP) (Figs. 3h and S3).

### Protein co-expression network analysis

**Fig. 4:**
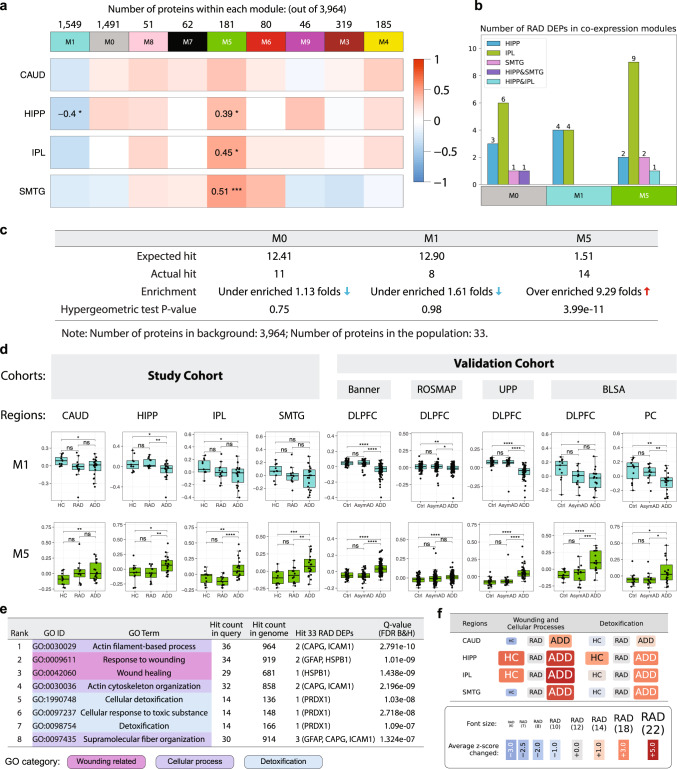
Consensus protein co-expression analysis and enrichment analysis results. **a** Consensus protein co-expression analysis identified 9 modules across four brain regions. Pearson correlation with two-sided *P*-values was used to evaluate the relationships between clinico-pathologic groups and eigenprotein expression. Exact *P*-values are provided in the Source Data file. **b** Three co-expression modules contained the 33 RAD DEPs. **c** The number of expected and observed RAD DEPs in each module, and enrichment analysis via two-sided hypergeometric test. **d** Module 1 and 5 eigenprotein expressions in HC, RAD, and ADD for the study set and in Ctrl, AsymAD, and ADD for external validation sets. The number of samples in the study cohort: HC = 11, RES = 12, ADD = 20. The number of samples in the external validation cohorts: Banner (Ctrl = 42, AsymAD = 45, ADD = 92), ROS/MAP (Ctrl = 78, AsymAD = 89, ADD = 162), UPP (Ctrl = 26, AsymAD= 20, ADD = 49), and BLSA (DLPFC: Ctrl = 11, AsymAD = 13, ADD = 17; PC: Ctrl = 13, AsymAD = 13, ADD = 19). For the boxplots, the interior horizontal line represents the median value, the upper and lower box edges represent the 75th and 25th percentile, and the upper and lower bars represent the 90th and 10th percentiles, respectively. **e** Top 3 enriched GO biological process categories in M5 and their enrichment analysis results. **f** Patterns of the change in M5 z-scores. Font sizes of clinico-pathologic groups reflect average z-score changes within the GO categories. Abbreviations: CAUD caudate, HIPP hippocampus, IPL inferior parietal lobule, SMTG superior and middle temporal gyrus, HC healthy control, RAD resilience to AD, ADD Alzheimer’s disease and dementia, DLPFC dorsolateral prefrontal cortex, PC precuneus, GO gene ontology. Source data are provided as a Source Data file.

To nominate related proteins that robustly can distinguish RAD, we expanded the analysis from individual proteins to protein modules using the established WGCNA algorithm^18^ to perform a consensus weighted protein co-expression network analysis on the 3964 proteins detected in all four regions (Fig. 4a). The resulting 9 co-expression modules were then used to estimate eigenproteins, which can be considered as the summary of a module’s overall protein expression^19^. As expected, the two isocortical regions had similar eigenprotein expression compared to the other two regions. The regions that undergo neurodegeneration in AD, HIPP, IPL, and SMTG, but not CAUD, showed significant positive correlations between clinico-pathologic groups and module (M) 5, while M1 in HIPP was negatively correlated with clinico-pathologic groups (Fig. 4a). The derived 9 co-expression modules displayed consistent patterns across external validation datasets (Fig. S17). Among individual-level data including age, sex, A score, B score, C score, and *APOE* ε4 allele, only age was positively correlated with M1 in both IPL and SMTG (Fig. S5). The 33 RAD DEPs were distributed across M0, M1, and M5 (Fig. 4b). Specifically, M0 contained Aβ, AL1L1, AL1L2, ANM5, EGFR, IF5, JMJD7, KHDR2, MK01, TICN3, and KS6A2; M1 contained BRI3B, AL1A1, FRIL, PDK2, SERC, PLCD3, WIPF2, and PBIP1; and M5 contained C04A, C04B, CAPG, HSPB1, K2C7, K2C8, CLUS, GFAP, FAAA, PRDX1, PA1B3, CMBL, ICAM1, and IRGQ. We performed Spearman correlation analysis for the 33 RAD DEPs within each region and summed the number of significantly correlated (Adjusted P-value < 0.05) proteins across all regions (Fig. S6). Of the 13 least correlated proteins, 11 of them were assigned to M0, including Aβ. Note that M0 represents a group of unassigned proteins.

A hypergeometric test evaluated the expression of the RAD DEPs among the consensus AD network modules (Fig. 4c). M5 was strongly enriched for expression of RAD DEPs (14 of 33 RAD DEPs; *P*-value = 3.99e−11), while M0 and M1 were not significantly enriched in RAD DEPs (M0 *P*-value = 0.75, M1 *P*-value = 0.98). Indeed, M5 was over-enriched in RAD DEPs by almost 10-fold compared to chance. M5 eigenprotein expression was then compared among the study set and external datasets (Fig. 4d). We found the same dementia-associated pattern (HC ≈ RAD < ADD) across all regions and all datasets, robustly validating that M5 eigenprotein expression was significantly greater in ADD compared to RAD or HC groups or compared to AsymAD or Ctrl groups (see also Figs. S11a and S17). Furthermore, a protein-protein interaction (PPI) network for M5 was constructed based on the STRING database v11.5^20^. Among the 181 proteins in M5, 177 primary genes were identified in the PPI network with 468 edges in total (average node degree = 5.29), indicating that PPI in M5 has significantly more interactions than expected by chance (*P* < 1.0e−16). Figure S8 shows the PPI network of M5 with experimentally determined interactions highlighted. Together, these results underscore that M5 is a robustly validated and densely co-expressed module of 181 proteins that distinguishes ADD from RAD despite their equivalent histopathologic burden of disease (Figs. S13–22).

### Enrichment analysis of M5 reveals RAD-associated processes

We performed gene ontology (GO) analysis using each M5 protein’s primary gene^21^ and identified the three top GO categories based on the branches in the ancestor chart: (i) Wounding Related (GO:0009611, GO:0042060), (ii) Cellular Process (GO:0030029, GO:0030036, GO:0097435), and (iii) Detoxification (GO:1990748 GO:0097237 GO:0098754). These top three GO categories contained the eight strongest GO terms according to enrichment P-values (Fig. 4e) and overlapped with sixty-nine of M5 proteins’ primary genes (Fig. S9b). Among these GO terms, Actin Filament-based Process (GO:0030029, *Q*-value = 2.791e−10) ranked highest with its descendant Actin Cytoskeleton Organization (GO:0030036, *Q*-value = 2.196e−09) ranked fourth. These two plus Supramolecular Fiber Organization (GO:0097435, *Q*-value = 1.324e−07) were grouped into the Cellular Process GO category (light purple) and shared two RAD DEPs: ICAM1 and CAPG. The Wounding Related GO category (dark purple), including Response to Wounding (GO:0009611, *Q*-value = 1.01e−09) and its subcategory Wound Healing (GO:0042060, *Q*-value = 1.438e−09), shared the RAD DEP HSPB1. The Detoxification GO category (light blue), including Cellular Response to Toxic Substance (GO:0097237, *Q*-value = 2.718e−08), Detoxification (GO:0098754, *Q*-value = 1.09e−07) and its descendant Cellular Detoxification (GO:1990748, *Q*-value = 1.03e−08) contained the RAD DEP PRDX1 (Fig. 4e). Thirteen out of 14 RAD DEPs detected in M5 were expressed higher in ADD than in RAD (Fig. S11b). Among these 13 RAD DEPs, 5 were included in M5’s top enriched GO terms and all were positively correlated with M5 eigenprotein. Spearman correlation for these 5 DEPs (CAPG, HSPB1, GFAP, PRDX1, ICAM1) showed mostly positive correlation with proteins present in both the GO terms and M5 (Fig. S11c).

Finally, a few regional and group patterns emerged when we calculated z-scores to assess whether the items in the different GO terms were up- or downregulated (Figs. 4e and S10). Wounding Related and Cellular Process are grouped because they always changed together with a consistent pattern of RAD several fold less than ADD in HIPP and isocortical regions and less pronounced group differences in CAUD. Detoxification repeated this general expression pattern with strong increase in ADD compared to RAD in HIPP and isocortical regions with less pronounced differences among groups in CAUD. In all GO categories for all regions, RAD more closely approximated HC than ADD (Fig. 4f).

### Enrichment analysis of M1 reveals AD-related processes

Although RAD DEPs were not significantly enriched in M1, we also performed gene ontology (GO) analysis using each of M1 proteins’ primary gene^21^ and identified four groups among the top 10 GO terms: (i) Synapse (GO:0099003, GO:0099536, GO:0099537, GO:0098916, GO:0007268); (ii) Energy (GO:0006091); (iii) Transport (GO:1902600, GO:0006812), and (iv) Cellular process (GO:0045333, GO:0007005) (Fig. S12). Of the 8 RAD DEPs in M1, two were included in these top 10 GO terms; AL1A1 was contained in generation of precursor metabolites and energy (GO:0006091, *Q*-value = 4.97e−75) and FRIL was contained in cation transport (GO:0006812, *Q*-value = 5.45e−71).

## Discussion

Our study focused on actual resilience to the clinical expression of dementia from AD, and because of this it had important design differences from previous proteomic studies of AD^3,22,23,4^. The most important difference is that we comprehensively evaluated brains to minimize confounding from latency in HC and from comorbidities in all groups. Exclusion of latent and comorbid diseases had the inevitable consequence of reducing the number of cases eligible for study; indeed only 43 of 737 eligible brain donations met our stringent criteria. In part to offset the impact of a relatively small number of high quality cases and controls, we expanded our study to include multiple brain regions involved or uninvolved by neurodegeneration. We only included intermediate or high ADNC in the matched RAD and ADD groups (*P* = 0.19), all with sufficient burden of AD to cause dementia (see Table S3) [1,40]. In contrast to the approach used by others, our focus on RAD excluded the large number of preclinical AD cases from our cohort; others instead have analyzed AsymAD, which is a mix of preclinical AD and RAD^3,4^. As far as we are aware, our focus on RAD is a unique study design.

Two-sided Student’s *t*-test with multiple testing adjustments revealed 33 RAD DEPs from comparisons across 21,160 unique protein-region pairs. The number of RAD DEPs after stringent selection and controlling for multiple testing gave a reliable and robust RAD protein signature. The strongest and most consistent RAD signal from our multiregional analysis was soluble Aβ expression, which tended to be in between HC and ADD in all regions, and uniquely Aβ expression in SMTG was significantly less in HC than RAD which in turn was significantly less than ADD. Indeed, soluble Aβ expression in SMTG was a major contributing variable to near complete separation of HC/RAD from ADD using PCA. These results suggest that although RAD and ADD groups were matched by the admittedly coarse tools for histopathologic scoring of ADNC, which includes ordinal ranking of insoluble Aβ plaques, lower tissue concentration of soluble Aβ might be a significant molecular feature of RAD. However, we recognize that our histopathologic matching is imperfect, and it is possible that the decreased soluble Aβ in RAD compared to ADD might be related in part to statistically insignificant variation in overall Aβ levels between these two groups. The soluble, lower molecular mass assemblies of Aβ are typically extracted with SDS (as done here) or urea, while extraction of higher molecular weight, insoluble aggregates of Aβ requires stronger chaotropics, like 70% formic acid^24^. Banner and ROS/MAP proteomic data for AsymAD, which contains both preclinical and RAD cases, validated our major finding that aligns readily with abundant experimental data showing that low molecular weight soluble aggregates of Aβ, forms not detected by histopathologic and PET methods that visualize insoluble Aβ fibrils, are concentration-dependent direct neuronal stressors and indirect neuronal stressors via glial cell activation^25,26^. Together, these cross-validating results support that lower tissue concentration of soluble Aβ in isocortex may be a molecular feature of RAD, plausibly resulting in reduced neuronal stress and injury. Most therapeutic antibodies that reduce brain Aβ target larger, less soluble forms of Aβ; so far these have largely failed in clinical trials^27^. In contrast, emerging agents like mAb158, also called BAN2401 or Lecanemab, binds to soluble Aβ species and has shown promising outcomes in initial clinical trials^28^. Our quantitative results from people resilient to ADD could provide a rough estimate of the extent to which soluble Aβ may need to be lowered in isocortical regions to suppress the clinical expression of severe cognitive impairment along the continuum of AD^29^.

Across all clinico-pathologic groups, we found that three RAD DEPs (Aβ, ICAM1, and IRGQ) were negatively correlated with TAU-195 only in the SMTG region, suggesting that these three RAD DEPs are increasing with TAU hyperphosphorylation in this brain region. These data underscore the well-described link between Aβ accumulation and TAU hyperphosphorylation, and suggest potential contributions of ICAM1 and IRGQ in this yet to be fully understood association.

We pursued other potential contributors to RAD through co-expression network analysis and observed that one module, M5 in HIPP, SMTG, and especially IPL, was significantly enriched in ADD resilience-associated proteins, a result validated in DLPFC using Banner and UPP, and both regions using BLSA data. Across our study and all validation sets, M5 had the pattern HC ≈ RAD < ADD, implying that increased expression of M5 proteins may be a molecular signature in isocortical and HIPP regions of progression to dementia. In contrast, M1 in HIPP was significantly underrepresented in RAD (HC ≈ RAD > ADD), a pattern validated in most external datasets and perhaps a consequence of the extensive neurodegeneration in this region that accompanies progression to dementia. Interestingly, Aβ was not a component of M5 or M1 but rather M0, meaning that it was not contained within a co-expression network despite its increasing tissue concentration being a distinguishing feature between RAD and ADD.

Functional insight into the 181 component proteins of strongly resilience-associated M5 by GO analysis yielded the three top categories including actin cytoskeleton organization, wound healing, and cellular detoxification. Actin filament dynamics are essential to dendrite and synapse formation and remodeling, and others have identified actin filament-based processes in enrichment analyses of AD^30^ and bipolar disorder^31^. Importantly, preserved synaptic density is a feature of resilience to AD, potentially linking our proteomic data with morphological data to highlight synaptic plasticity as a key compensatory feature of resilience^32^. Wound healing and detoxification are complex responses to injury that showed a similar pattern of expression as actin filament dynamics such that these three biological processes, which represent compensatory change and response to injury, were several fold greater in ADD than RAD in the three regions undergoing neurodegeneration. The lower response to injury in HIPP and isocortical regions of RAD aligns well with our results showing lower soluble Aβ concentration in these regions as well as with the extensively described mechanisms of Aβ induced injury to neurons through both direct and indirect mechanisms^33,34^. Together, these results suggest that RAD is a state of equivalent histopathologic features but lower soluble Aβ and less injury than ADD. In addition, we note that none of the M1 RAD DEPs were included in the Synapse GO terms unlike what others have published for AsymAD^3,4,35,36^ perhaps because of our focus on RAD rather than AsymAD or our careful exclusion of co-morbidities. Further, our results suggest that RAD is multifactorial and future studies will need to determine whether modulation of some or all of actin cytoskeleton organization, wound healing, and cellular detoxification are necessary to achieve resilience.

Of the 33 RAD DEPs identified, M5 contained 14 and a few of these deserve specific mention. CLUS, or clusterin, is also known as apolipoprotein (apo) J. Variants in the CLUS gene have been repeatedly associated with the risk of AD. Like the closely functionally related APOE protein whose concentration was strongly positively correlated, CLUS plays a major role in lipid transport in brain where it critically supports synaptic remodeling and repair, and modulates innate immune responses involving the RAD DEPs CO4A and CO4B. ICAM1 is a master regulator of inflammation and injury resolution and was a major contributing variable in multiple brain regions to the near complete separation of HC/RAD from ADD by PCA^37^. Immune-mediated neuronal injury is widely supported as a major contributor to AD pathogenesis, and variants in the gene encoding ICAM1 have been associated with risk of AD^33^. The platelet-activating factor acetylhydrolase isoform 1B complex, of which PA1B3 is a subunit, is broadly expressed across neuronal cell types and is important in human brain development, including neuron migration^38,39^. Also known as PAFAH1B3, this protein was recently shown by elegant proteomic work of others investigating Ctrl, AsymAD, and ADD samples from ROS/MAP to reside at the center of a MAPK/metabolism module of proteins associated with both amyloid plaques and neurofibrillary tangles^3^.

Intriguing anatomical differences also were observed in our data. The CAUD subserves motor, cognitive, and behavioral functions, and has been proposed as a site for temporary functional compensation in AD^40,41^. Neither our differential expression analysis nor consensus protein expression analysis identified significant changes in CAUD, suggesting that like neurodegeneration the proteomic changes in this region in AD are minimal. Rather both types of analysis localized proteomic changes of RAD to HIPP and isocortex, further suggesting that proteomic changes associated with RAD are unlikely to be nonspecific brain changes that accompany systemic impacts of dementia. The two isocortical regions investigated, SMTG and IPL, had only one overlapping RAD DEP, CAPG. Murine *capg* expression was shown recently to be among a small set of disease-associated microglia genes uniquely upregulated by *APOE* ε4 in preclinical models of AD^42^. Resilience-associated proteomic changes were largely non-overlapping in IPL or SMTG, and were only partially validated in prefrontal cortex or precuneus from external datasets, perhaps indicating regional variation in isocortical contributions to RAD. Although the RAD DEPs displayed regional variation, co-expression analysis showed a consistent pattern for HIPP, IPL, and SMTG. Finally, when comparing up or down regulation of Wounding Related and Cellular Process GO terms, CAUD and SMTG displayed the intriguing pattern of down regulation in RAD vs. HC and upregulation in RAD vs. ADD, while HIPP and IPL showed upregulation in both group comparisons. Although the significance of these region-by-group interactions are not clear, they underscore the likely regional variation in molecular mechanisms of RAD.

Often called by different names, the concept of resilience—individuals with high levels of disease burden yet remain cognitively normal—has been widely documented in the neuroimaging, biomarker, and neuropathology literature^2,43^. While low level disease in preclinical individuals is pathogenic^44^, it is the mismatch of high pathologic burden with normal cognition that defines RAD. We drew on the NIA-supported Collaboratory on Research Definitions for Cognitive Reserve and Resilience, whose goal was to develop operational definitions, research guidelines, and data sharing platforms to facilitate communication among emerging research across the globe^45^. Although we can distinguish between preclinical AD and RAD based on ADNC at the time of death, it is impossible to know if someone in either of these categories would have been diagnosed with dementia, or if a case of preclinical AD might have advanced to RAD, had the person lived longer. One approach to address this difficult issue is to reference clinico-pathologic categories to the individuals’ ages, assuming that older individuals would have lower likelihood of theoretical future category change. Here, we investigated RAD in individuals who lived to 78–98 years of age (average 89). The average age of the asymptomatic AD groups in three of the comparison studies (Banner, ROS/MAP, and BLSA) was 86–87 years, while the average age of the fourth asymptomatic AD comparison study was only 70 years (UPP).

We have shown previously that one factor underlying apparent resilience is clinical misassignment due to incomplete information on co-morbidities^8^. Our work here removed confounding by co-morbidities and revealed possible protein contributors to this discordant state for AD. Our findings suggest lower soluble Aβ tissue concentration as a potential signature for RAD. This lower concentration could result from reduced AD-dependent processes or from AD-independent processes. Indeed, it is likely that multiple contributors to preserving cognitive ability despite high levels of disease burden, including molecular, cellular, and neural circuits, are yet to be discovered for AD and other diseases that commonly conspire to cause cognitive impairment in older individuals.

This study has several limitations. First, because we used stringent criteria for cohort enrollment, a relatively small group of 43 high-quality cases out of 737 eligible was assembled to focus on RAD. Second, bulk tissue proteomics lacks cell-type specificity and likely obscures important cell-specific changes. In light of our results, we hope future studies will focus on single-cell analysis to further disclose cell type-specific changes in RAD. Third, like all bottom-up proteomic studies, we are unable to distinguish Aβ generated in vivo from trypsin digestion products of APP that generate the Aβ fragment ex vivo. However, we note that our proteomic Aβ measurements based on the peptides HDSGYEVHHQK and LVFFAEDVGSNK were significantly correlated with A score and C score, both histologic measures of Aβ accumulation. Furthermore, other groups have demonstrated that the tryptic peptides from APP spanning the Aβ region are a valuable surrogate for amyloid levels in the brain^23,46^. Lastly, restricted by the availability of stringently defined cases, we conducted independent validation using external datasets that most closely approximated ours. We hope our results motivate others to try to validate our findings by creating an animal model or developing similarly stringently defined clinico-pathological cohorts.

In conclusion, we have undertaken an extensive proteomic analysis of carefully annotated human brain regions to determine molecular features of RAD. When compared to ADD, our validated results show that lower tissue concentration of soluble Aβ in isocortical regions as well as lower expression of actin filament-based processes and cellular detoxification/repair in isocortex and hippocampus are characteristic of RAD. Combined with the results of others, our study suggests that people with RAD have lower disease-specific injury, perhaps from less soluble Aβ, and thereby an appropriately limited response to injury. These results provide insights into the molecular features of RAD and suggest potential therapeutic strategies to limit the clinical progression to Alzheimer’s disease and dementia.

## Methods

### Clinico-pathologic groups

This research was ethically approved by the University of Washington Institutional Review Board (IRB) and the Stanford University IRB. All study cohort participants provided informed consent under protocols approved by these IRBs. Sex was considered in the study design and was determined based on self-report (male *N* = 24 (55.81%), female *N* = 19). Cognitive diagnosis of dementia or not dementia was made using DSM-IVR criteria; an initial provisional diagnosis of dementia was followed one year later with a confirmed diagnosis of dementia. Starting with 737 brain donations, a total of 340 research brain donations that had been dissected and flash frozen within 8 h (mean ± SD = 4.4 ± 1.3 h) of death. Among them, 43 cases met eligibility criteria for rigorously defined clinico-pathologic groups (Table S4): (i) Healthy controls (HC, *N* = 11) had neuropsychological test results in the upper quartile for the cohort at their last visit within 2 years of death, did not have AD neuropathologic change (ADNC) according to NIA-AA guidelines, and had clinically insignificant (none/low) pathologic changes of VBI, LBD, HS, or LATE^1,47,48^; (ii) Cognitive resilience to AD (RAD, *N* = 12) had neuropsychological test results in the upper quartile for the cohort at their last visit within 2 years of death, had intermediate or high level ADNC according to NIA-AA guidelines, and had none/low pathologic changes of VBI, LBD, HS, or LATE^1,47,48^; and (iii) AD dementia (ADD, *N* = 20) were diagnosed with dementia during life, had intermediate or high level ADNC according to NIA-AA guidelines, and had none/low pathologic changes of VBI, LBD, HS, or LATE^1,47,48^. Less common neurodegenerative diseases, including FTLD-tau and FTLD-TDP also were excluded following consensus guidelines^1^. In addition to hematoxylin and eosin plus luxol fast blue histochemical staining for assessment of vascular brain injury (VBI) and hippocampal sclerosis (HS), immunohistochemistry for Aβ plaques (anti-b-Amyloid, 6E10, Biolegend, cat#803002, dilution 1:1000), and neurofibrillary degeneration (PHF-Tau, AT8, ThermoScientific, cat#MN1020, dilution 1:1000) was performed in specified regions^47^. Antibodies to phospho-TDP-43 (anti-pTPD-43, S409/410, Millipore, cat#MABN14, dilution 1:300) and phospho-alpha-synuclein (Anti-Alpha-synuclein, ps129, Abcam, cat#ab51253, dilution 1:1000) also were used in specified regions^47,49^. All cases were originally evaluated by one of the co-authors who is a board-certified neuropathologist (C.L., C.D.K., or T.J.M.) and all those selected for inclusion in the study were reviewed by T.J.M. Importantly, we excluded from all clinico-pathologic groups cases with low-level ADNC according to NIA-AA guidelines^1,47^. We have shown previously insignificant interval change in diagnosis of not dementia over 2 years between last research evaluation and death for individuals who had neuropsychological test results in the upper quartile for the cohort^50^; all HC and RAD participants had <2 years between last evaluation and death with an average interval of 352 days. Criteria for including only none/low levels of the four prevalent comorbidities that do not significantly contribute to the risk of dementia were: (i) for VBI: no territorial or lacunar infarcts, no hemorrhages, <2 microinfarcts/microhemorrhages^51^, (ii) for LB: none or amygdala only^52^, (iii) for LATE-NC: none or amygdala only^53^, and (iv) no hippocampal sclerosis in the unilateral hippocampus available for histopathologic analysis.

### Sample preparation and proteomic analysis

Samples were dissected to be free of adjacent white matter by visual inspection^10^. Two 25 μm thin frozen sections were maintained at −80 °C from four brain regions: caudate nucleus (CAUD, *N* = 38), hippocampus (HIPP, *N* = 41), inferior parietal lobule (IPL, *N* = 38), and superior and middle temporal gyrus (SMTG, *N* = 38) for a total of 155 samples (30 cases were matched across all four regions; Fig. S1). SDS protein extraction was performed as previously described by us^9^ and presented in the Supplementary Methods.

The Skyline documents, raw files for quality control and DIA data are available at Panorama Public https://panoramaweb.org/ADBrainCleanDiagDIA.url. ProteomeXchange ID: PXD034525. Access URL: http://proteomecentral.proteomexchange.org/cgi/GetDataset?ID=PXD034525. Metadata of the study cohort is available in Supplementary Information.

Peptide data was acquired by DIA mass spectrometry (DIA-MS) based on the methodology described elsewhere^10,54,55^. Protein levels were calculated by summing peptide abundances after performing protein inference and parsimony^56^. After ${{{{{\rm{log}}}}}}_{2}(x)$ transformation, median normalization was performed to adjust for minor sources of variability that are difficult to either control or predict, followed with batch correction to remove the effect of different experimental batches. In external datasets, for proteins with two or more isoform identifiers, we kept the expressions with maximum read counts.

### Protein extraction

Protein was extracted from the frozen brain sections by resuspension in 120 μl of lysis buffer containing 5% SDS, 50 mM triethylammonium bicarbonate (TEAB), 2 mM MgCl_2_, 1X HALT phosphatase and protease inhibitors. The samples were vortexed and briefly sonicated at setting 3 for 10 s with a Fisher sonic dismembrator model 100. A Barocycler 2320EXT (Pressure Biosciences Inc.) was used to further homogenize 30 μl of the brain lysate in a microtube capped with a micropestle for a total of 20 min at 35 °C with 30 cycles of 20 s at 45,000 psi followed by 10 s at atmospheric pressure. The concentration of protein was determined using a BCA assay (Pierce). A process control of 800 ng of yeast enolase protein (Sigma) was added to 50 µg of brain homogenate which was then reduced with 20 mM DTT and alkylated with 40 mM IAA. The samples were then prepared for S-trap column (Protifi) binding by the addition of 1.2% phosphoric acid and 350 μl of binding buffer (90% Methanol, 100 mM TEAB). The acidified sample was bound to column incrementally, followed by 3 wash steps with binding buffer to remove SDS and 3 wash steps with 50:50 methanol:chloroform to remove lipids and a final wash step with binding buffer. Each wash step included 1 min centrifugation at 4000 × *g*. The digestion buffer of trypsin (1:10) in 50 mM TEAB was then added to the S-trap column for digestion at 47 °C for 1 h. First hydrophilic peptides were eluted using a 50 mM TEAB buffer with 1 min of centrifugation at 4000 × *g*. Then hydrophobic peptides were eluted with a solution of 50% acetonitrile in 0.2% formic acid followed by 1  min of centrifugation at 4000 × *g*. Elutions were pooled, speed vacuumed and resuspended in 0.1% formic acid.

We used S-trap sample preparation because it resulted in protein digestion from brain samples with the greatest robustness and quantitative precision in empirical analyses. Brain is lipid rich, and the tissue sections used contained optimal cutting temperature (OCT) compound. OCT compound is particularly problematic as it causes peptide signal suppression in electrospray. The use of 5% SDS was selected because it is predicated for use with S-trap (Protifi) digestions. The proteins were solubilized using a combination of 5% SDS and high pressure barocycling (45,000 PSI).

The samples injected for DIA-LC/MS contained one µg of total protein with 16 ng of enolase process control plus 150 fmol of a heavy labeled Peptide Retention Time Calibrant (PRTC) mixture (Pierce) used as a peptide process control. All samples and references from a batch are equally represented in the library pools. For example, a batch library pool consists of the 14 samples from the batch and two references. System suitability injections contain 150 fmol of PRTC and BSA which is used to assess the system before and during analysis. Four of these system suitability runs are analyzed prior to any sample analysis and then after every six sample runs another system suitability run is analyzed.

Each sample was loaded onto a 30 cm fused silica picofrit (New Objective) 75 µm column and 3.5 cm 150 µm fused silica Kasil1 (PQ Corporation) frit trap loaded with 3 µm Reprosil-Pur C18 (Dr. Maisch) reverse-phase resin analyzed with a Thermo Easy-nLC 1200. Liquid chromatography (LC) buffer A was 0.1% formic acid in water and LC buffer B was 0.1% formic acid in 80% acetonitrile. The 40-min system suitability gradient consists of a 0–16% B in 5 min, 16–35% B in 20 min, 35–75% B in 1 min, 75–100% B in 5 min, followed by a wash of 9 min and a 30-min column equilibration. The 110-min sample LC gradient consists of a 2–7% for 1 min, 7–14% B in 35 min, 14–40% B in 55 min, 40–60% B in 5 min, 60–98% B in 5 min, followed by a 9-min wash and a 30-min column equilibration. Peptides were eluted from the column with a 50 °C heated source (CorSolutions) and electrosprayed into a Thermo Orbitrap Fusion Lumos Mass Spectrometer with the application of a distal 3 kV spray voltage. For the system suitability analysis, a cycle of one 120,000 resolution full-scan mass spectrum (350–2000 *m*/*z*) followed by a data-independent MS/MS spectra on the loop count of 76 data independent MS/MS spectra using an inclusion list at 15,000 resolution, AGC target of 4e5, 20 millisecond (ms) maximum injection time, 33% normalized collision energy with an 8 m/z isolation window. For the sample digest, first a chromatogram library of 6 independent injections is analyzed from a pool of all samples within a batch. For each injection a cycle of one 120,000 resolution full-scan mass spectrum with a mass range of 100 *m*/*z* (400–500, 500–600, 600–700, 700–800, 800–900, 900–1000 *m*/*z*) followed by a data-independent MS/MS spectra on the loop count of 26 at 30,000 resolution, AGC target of 4e5, 60 ms maximum injection time, 33% normalized collision energy with a 4 *m*/*z* overlapping isolation window. The chromatogram library data is used to quantify proteins from individual sample runs. These individual runs consist of a cycle of one 120,000 resolution full-scan mass spectrum with a mass range of 350–2000 *m*/*z*, AGC target of 4e5, 100 ms maximum injection time followed by a data-independent MS/MS spectra on the loop count of 76 at 15,000 resolution, AGC target of 4e5, 20 ms maximum injection time, 33% normalized collision energy with an overlapping 8 m/z isolation window as described previously^54,57^. Operation of the mass spectrometer and LC solvent gradients are controlled by the ThermoFisher Xcalibur (version 3.1.2412.24) data system.

Thermo RAW files were converted to mzML format using Proteowizard (version 3.0.20064) using vendor peak picking and demultiplexing with the settings of “overlap_only” and Mass Error = 10.0 ppm. On column chromatogram libraries were created using the data from the six gas phase fractionated “narrow window” DIA runs of the pooled reference. These narrow windows were analyzed using EncyclopeDIA (version 1.4.10) with the default settings (10 ppm tolerances, trypsin digestion, HCD b- and y-ions) of a Prosit predicted spectra library based on the UniProt human canonical FASTA (January 2021). The results from this analysis from each brain region were saved as a “Chromatogram Library” in EncyclopeDIA’s eLib format where the predicted intensities and iRT of the Prosit library were replaced with the empirically measured intensities and RT from the gas phase fractionated LC-MS/MS data. The “wide window” DIA runs were analyzed using EncyclopeDIA (version 1.4.10) requiring a minimum of 3 quantitative ions and filtering peptides with *Q*-value ≤ 0.01 using Percolator 3.01. After analyzing each file individually, EncyclopeDIA was used to generate a “Quant Report” which stores all the detected peptides, integration boundaries, quantitative transitions, and statistical metrics from all runs in an eLib format. The Quant Report eLib library is imported into Skyline (daily version 22.2.1.278) with the human UniProt FASTA as the background proteome to map peptides to proteins, perform peak integration, manual evaluation, and report generation.

### Derivation of 33 RAD DEPs

We conducted two-sided Student’s *t*-tests for RAD vs. HC, RAD vs. ADD, and ADD vs. HC; each comparison was run for 21,160 unique protein-region pairs. We observed 7723 (12.17%) protein comparisons with *P*-values < 0.05 (1767 for RAD vs. HC, 2658 for RAD vs. ADD, and 3298 for ADD vs. HC). After controlling for multiple hypothesis tests using the Benjamin-Hochberg procedure (FDR = 0.05), we identified 85 significant protein comparisons (adjusted *P*-value < 0.05). Of the 85 significant protein comparisons (Fig. 2c), 76% (*N* = 65), were unique indicating that most group differences in protein expression were region-specific. The regional distribution of the 85 DEPs was coded for significant paired group differences: 43 had significantly different expression between ADD and HC, including only 3 in CAUD (Aβ, MT3, PA1B3) with the Aβ result in ADD CAUD confirming our group assignments. Importantly, the 42 preliminary RAD DEPs with significantly different expression between RAD and HC or between RAD and ADD were restricted only to HIPP and isocortical (IPL and SMTG) regions. Nine of the 42 preliminary RAD DEPs had significantly different expression when comparing RAD vs. HC (3 RAD < HC and 6 RAD > HC), and 33 had significantly different expression when comparing RAD vs. ADD (3 RAD > ADD and 30 RAD < ADD). With Aβ and CAPG expression significantly different in two regions, and Aβ in SMTG and IF5 in HIPP overlapping between RAD vs. HC and RAD vs. ADD, there were 38 unique preliminary RAD DEPs. Five of the 38 unique preliminary RAD DEPs were not detected in all four regions (Fig. 3a); these five were excluded from further analysis, yielding 33 RAD DEPs for detailed analysis.

### Handling missing values

When performing univariate analysis, proteins for which all expressions are missing were removed. If a protein has less than 3 available regional sample expressions, the Student’s *t*-test and Spearman correlation will not be performed for that protein (use missing value instead). When performing low dimensional visualization and consensus protein co-expression analysis, missing protein expressions were imputed by mean values from other individuals within a certain brain region. When validating protein co-expression modules with external cohorts, proteins were either discarded if all values were unavailable or imputed by mean values from other individuals if available.

### Consensus protein co-expression module analysis

For consensus protein co-expression module analysis, we adopted the WGCNA algorithm and chose soft power = 7 according to Fig. S4b. And by default, we chose deep split = 2, minimum module size = 30, and merging cut height = 0.25. The consensus co-expression module analysis takes all four brain regions into account, and developed a single set of modules. After consensus merging, module 2 was merged into module 1, and module 0 represents a group of unassigned proteins, resulting 9 modules. Based on categorical groups conditions (HC: 0, RAD: 1, ADD: 2), Pearson correlation was performed to evaluate the relationships between clinico-pathologic groups and expression of the 9 eigenproteins, followed with P-value adjustment (FDR cut-off = 0.05).

### Gene ontology enrichment analysis

Gene ontology (GO) enrichment analysis was carried out by ToppGene suite (version 2022-03-28. 20,669 genes in category)^21^. Protein’s primary gene was derived according to UniProtKB (2022_01) database. Ancestor chart was constructed according to AmiGO2 Gene Ontology database^58^. Z-scores were calculated based on number of up/down regulated proteins from hit count in query:

1 $${{{{{\rm{zscore}}}}}}=\frac{({N}_{{{{{{\rm{up}}}}}}}-{N}_{{{{{{\rm{down}}}}}}})}{\sqrt{{N}_{{{{{{\rm{hitcount}}}}}}}}}$$

Up and down regulation was based on ${{{{{\rm{lo}}}}}}{{{{{{\rm{g}}}}}}}_{2}$ fold change. If a z-score > 0 on RAD vs HC, then the associated GO term is more likely to be increased when RAD > HC^59^. We set z-score = ±1 as the cut-off threshold to determine the up/down regulation signal of a GO term. Patterns of the change in M5 z-scores were described in fontsize = $12+2\times ({{{{{\rm{zscor}}}}}}{{{{{{\rm{e}}}}}}}_{{{{{{\rm{avg}}}}}}})$, where ${{{{{\rm{zscor}}}}}}{{{{{{\rm{e}}}}}}}_{{{{{{\rm{avg}}}}}}}$ is the average change of z-score within a GO category when comparing RAD to other clinico-pathologic groups.

### Validation with external datasets

For external validation, 179 individuals (Ctrl = 42, AsymAD = 45, ADD = 92) were collected from Banner Sun Health Research Institute (Banner)^12^ in dorsolateral prefrontal cortex region (DLPFC, Brodmann area 9), followed with batch effects removal via ComBat^60^. 329 individuals (Ctrl = 78, AsymAD = 89, ADD = 162) were collected from Religious Orders Study and Rush Memory and Aging Project (ROS/MAP)^11^ in DLPFC region (TMT quantitation, version: 03/22/2022, SwissProt and TrEMBL human protein db 2015, median polish corrected relative reporter abundance, followed with ${{{{{\rm{lo}}}}}}{{{{{{\rm{g}}}}}}}_{2}(x)$ transformation), and 95 individuals (Ctrl = 26, AsymAD = 20, ADD = 49) were collected from the UPenn Proteomics study (UPP)^13^ in DLPFC region (label-free quantitation, version: 03/22/2022, median polish ratio over global internal standard (GIS), batch corrected relative reporter abundance, followed with ${{{{{\rm{lo}}}}}}{{{{{{\rm{g}}}}}}}_{2}(x)$ transformation). In addition, 41 individuals (Ctrl = 11, AsymAD = 13, ADD = 17) in DLPFC region and 45 individuals (Ctrl = 13, AsymAD = 13, ADD = 19) in precuneus region (PC, Brodmann area 7) were collected from Baltimore Longitudinal Study of Aging (BLSA)^14^. Although these external datasets were the most closely related, there were potentially important differences in clinico-pathologic group assignments between our study and these four external datasets.

1. Controls: By expert consensus guidelines from NIA-AA, our HC group was free of ADNC and clinically significant levels of the four other commonly comorbid diseases, while the approach used by the external datasets permitted low level ADNC in the control group and did not exclude VBI, LBD, HS, and LATE-NC from the control group.
2. RAD vs. AsymAD: Our approach only included intermediate or high ADNC in the RAD group, while the approach used by others permitted low level ADNC in the AsymAD group (preclinical AD)^3,4^. Intermediate or high level ADNC is sufficient to cause dementia^1,47^, meaning that our approach focused on RAD while AsymAD is a mix of preclinical AD and RAD cases (see Table S2).
3. Apparent vs. actual resilience: Neuropathologic assessment of VBI, LBD, HS, and LATE-NC was not included in the earlier proteomic studies, so these diseases not only are unknowingly present in the control group (vide supra), but apparent resilience cannot be distinguished from actual RAD without evaluation of all five diseases^8^.
4. Dementia was classified differently among all datasets: for ROS/MAP, dementia was clinical cognitive diagnosis summary at last visit (dcfdx_lv) > 1^3,4^, which includes mild cognitive impairment and dementia; for Banner, dementia was last MMSE < 24^3,4^; for BLSA and UPP dementia was AD diagnosis code^3,4^.

Our more stringent criteria fall within those used by others, meaning that our HC was a subset of external Ctrl, our RAD was a subset of external AsymAD, and our ADD was a subset of external ADD. When we applied our more stringent criteria for ADNC to the external datasets, between about one-quarter and one-half of cases were excluded. If the other four comorbidities had been evaluated, then even more external cases and controls would have been excluded, underscoring the reality of the limited availability of high-quality samples to investigate RAD. We struck the balance of comparing our results for HC, RAD, and ADD to Ctrl, AsymAD, and ADD of the most closely related external datasets as the best available external validation.

### Statistics and reproducibility

This study was designed to use computational approaches to analyze mass spectrometry-based proteomics data for resilience to Alzheimer’s disease in human brain. No statistical method was used to predetermine sample size, and no data were omitted from the analyses. All of the samples included in our study were utilized in the experiments. Since the cohort had already been established, there was no subsequent randomization during the statistical analysis. Researchers were blinded to clinico-pathologic group assignment until after collection of proteomic data. In statistical analysis, blinding was not performed after the collection of proteomic data, as cohort and label of each sample had been defined. All protein expressions were ${{{{{\rm{lo}}}}}}{{{{{{\rm{g}}}}}}}_{2}(x)$ transformed before statistical analyses. For differentially expressed proteins analysis, two-sided Student’s *t*-tests were performed with P-values adjusted for multiple comparisons. For correlation analysis, Spearman’s rank correlation method was performed to evaluate the protein expressions. In consensus weighted protein co-expression analysis, clinico-pathological groups and other factors given module eigenproteins were analyzed by Pearson correlation. For analyzing the concentration of RAD DEPs among co-expressed modules and calculating *Q*-values in gene ontology enrichment analysis^21^, hypergeometric test^61^ with probability mass function

2 $${{{{{\rm{Pr }}}}}}\left(X=k\right)=\frac{\left({K}\atop{k}\right)\left({N-K}\atop{n-k}\right)}{\left({N}\atop{n}\right)}$$

was adopted, where the binomial coefficient is defined as

3 $$\left({N}\atop{k}\right)=\frac{n!}{k!\left(n-k\right)!}.$$

In above equation, $N$ is the population size (total number of genes in the background), $K$ is the number of hits in the targeted gene list, $n$ is the number of draws in the protein list of interests, and $k$ is the number of observed successes (The total number of mutual proteins in both protein list of interests and the targeted protein list).

For low-dimensional visualization, principal component analysis (PCA) was adopted. Source code to derive the experiment results can be found in Github: https://github.com/huangzhii/RAD/. All experiments were performed in Macintosh OS v12.5, with the Apple M1 Pro CPU (10 threads) and 16 GB memory. All differential expression analyses and correlation analyses were performed in Python v3.9.7, with the following packages: pandas v1.4.2, numpy v1.19.5, matplotlib v3.5.1, mpl_toolkits v1.2.1, seaborn v0.11.2, scipy v1.8.0, scikit-learn v0.24.2, statsmodels v0.13.2, umap v0.5.2, combat v0.3.2, multiprocess v0.70.12.2. All consensus co-expression analysis was performed in R v4.1.2, with the following packages: WGCNA v1.70.3, stringr v1.4.0, ggpubr v0.4.0, ggplot2 v3.3.5, dplyr v1.0.8, FactoMineR v2.4, factoextra v1.0.7.

### Reporting summary

Further information on research design is available in the Nature Portfolio Reporting Summary linked to this article.

## Supplementary information

Supplementary Information

reporting summary

## Data Availability

The Skyline documents, raw files for quality control, and DIA data used in this study are available at Panorama Public with the following link: https://panoramaweb.org/ADBrainCleanDiagDIA.url. The proteomics data generated in this study have been deposited in the ProteomeXchange database under the access code: PXD034525 with the following link: The external validation data are available and can be obtained at https://www.synapse.org/. Specifically, Banner (https://www.synapse.org/#!Synapse:syn7170616), ROS/MAP (https://www.synapse.org/#!Synapse:syn3219045), UPP (https://www.synapse.org/#!Synapse:syn17009177), and BLSA (https://www.synapse.org/#!Synapse:syn3606086). UniProtKB (2022_01) database can be reached at https://www.uniprot.org/release-notes/2022-02-23-release. The experimental results generated in this study are provided in the Supplementary Information and Source Data file. Source data are provided with this paper.

## Source data

Source Data

## Code availability

Source code is available at Github: https://github.com/huangzhii/RAD/. Python and R codes were used to study and analyze the proteomics data.
