# Supplementary material for: Brain proteomic analysis implicates actin filament processes and injury response in resilience to Alzheimer’s disease
Source: Nat Commun. 2023 May 12;14:2747. doi: 10.1038/s41467-023-38376-x (PMC10182086; doi:10.1038/s41467-023-38376-x)
Supplement: Supplementary file 1 — Supplementary Information [file 41467_2023_38376_MOESM1_ESM.pdf]

Supplementary Information

Supplementary Tables

Table S1. Characteristics of study participants and samples.

		Clinico-pathologic groups			Total
		**HC**	**RAD**	**ADD**	
Cohort summary	Unique number of individuals	11	12	20	43
	Unique number of samples	37	44	74	155
	Age (yr; median, min-max)	88 (73 - 95)	90 (78 - 98)	85 (59 - 100)	88 (59 - 100)
	Sex (% Male)	54.55%	50.00%	60.00%	55.81%
Brain regions	CAUD (number of samples)	10	10	18	38
	HIPP (number of samples)	10	11	20	41
	IPL (number of samples)	8	12	18	38
	SMTG (number of samples)	9	11	18	38

		Number of proteins	Number of peptides
Regional proteomics data	CAUD	4,635	26,123
	HIPP	5,117	31,863
	IPL	6,099	42,492
	SMTG	5,851	40,342
	Total	7,115 (unique), 3,964 (shared)	56,073 (unique), 19,218 (shared)

Abbreviations: HC for healthy control, RAD for resilience to AD, ADD for Alzheimer's disease and dementia, CAUD for Caudate, HIPP for Hippocampus, IPL for Inferior parietal lobule, and SMTG for superior and middle temporal gyri.

Table S2. The criteria for clinicopathologic groups for the current study and datasets used for external validation are summarized. Note that unlike the external validation datasets, the current study explicitly excluded*: cases with any LB or LATE-NC other than amygdala, >2 microinfarcts or microhemorrhages, any territorial or lacunar infarcts, any hemorrhages, any HS, and any other neuropathologic features of the disease.

<table>
<tr><th colspan="2" rowspan="3">Clinico-pathologic groups</th><th colspan="4">Clinical</th></tr>
<tr><th colspan="2">Dementia: No</th><th colspan="2">Dementia: Yes</th></tr>
<tr><th>Current Study</th><th>External Validations</th><th>Current Study</th><th>External Validations</th></tr>
<tr><td rowspan="3">Pathological</td><td>Not ADNC</td><td>Healthy Control (HC)*</td><td rowspan="2">Control (Ctrl)</td><td colspan="2" rowspan="2">Excluded
(Causes of dementia other than AD)</td></tr>
<tr><td>Low ADNC</td><td>Excluded
(Preclinical AD)</td></tr>
<tr><td>Intermediate or High ADNC</td><td>Resilient to AD (RAD)*</td><td>Asymptomatic (Asym) AD</td><td>ADD*</td><td>ADD</td></tr>
</table>

Table S3. The Chi-square test compares the intermediate and high Alzheimer's disease neuropathologic change (ADNC) between RAD and ADD groups.

	**Intermediate ADNC**	**High ADNC**
RAD group	5	7
ADD group	4	16
Chi-square test P-value = 0.19.		

Table S4. Cohort metadata and clinico-pathologic groups. Note: *DSM-IVR criteria. ^NIA-AA ADNC score. Criteria for including only clinically insignificant levels of four common comorbidities were: (i) No territorial or lacunar infarcts, no hemorrhages, <2 microinfarcts; (ii) No LBs or LB in amygdala only; (iii) No LATE-NC or LATE-NC in amygdala only; (iv) No hippocampal sclerosis in the unilateral hippocampus available for histopathologic analysis. Additional potential identifiers such as APOE ε4 allele and postmortem interval (PMI) were collected but are not shown here.

Individual	Group	Age	Sex	Diagnosis*	ADNC Score^	A Score	B Score	C Score	Thal phase	Braak stage	CERAD score	CAUD	HIPP	IPL	SMTG
1	HC	73	Male	No dementia	Not ADNC	1	1	0	1 (A1)	II	No neuritic plaques (C0)	yes	yes	no	no
2	HC	78	Male	No dementia	Not ADNC	1	0	0	1	0	No neuritic plaques (C0)	yes	no	no	no
3	HC	79	Female	No dementia	Not ADNC	1	1	0	1	II	No neuritic plaques (C0)	yes	yes	no	yes
4	HC	84	Male	No dementia	Not ADNC	1	1	0	2	I	No neuritic plaques (C0)	yes	yes	yes	yes
5	HC	86	Male	No dementia	Not ADNC	1	2	0	2 (A1)	III	No neuritic plaques (C0)	yes	yes	yes	yes
6	HC	88	Female	No dementia	Not ADNC	0	1	0	0 (A0)	II	No neuritic plaques (C0)	yes	yes	yes	yes
7	HC	88	Female	No dementia	Not ADNC	1	2	0	2	III	No neuritic plaques (C0)	yes	yes	yes	yes
8	HC	89	Male	No dementia	Not ADNC	0	1	0	0 (A0)	I	No neuritic plaques (C0)	yes	yes	yes	yes
9	HC	90+	Male	No dementia	Not ADNC	1	2	0	2	III	No neuritic plaques (C0)	yes	yes	yes	yes
10	HC	90+	Female	No dementia	Not ADNC	1	2	0	2	IV	No neuritic plaques (C0)	no	yes	yes	yes
11	HC	90+	Female	No dementia	Not ADNC	3	2	0	4 (A3)	IV	No neuritic plaques (C0)	yes	yes	yes	yes
12	RAD	78	Male	No dementia	Intermediate	3	2	2	4	III	Moderate (C2)	no	yes	yes	no
13	RAD	79	Male	No dementia	Intermediate	3	2	2	5	III	Moderate (C2)	yes	no	yes	yes
14	RAD	86	Female	No dementia	Intermediate	3	2	2	4	IV	Moderate (C2)	yes	yes	yes	yes
15	RAD	87	Male	No dementia	High	3	3	3	5 (A3)	VI	Frequent (C3)	yes	yes	yes	yes
16	RAD	89	Female	No dementia	High	2	3	3	3 (A2)	V	Frequent (C3)	yes	yes	yes	yes
17	RAD	89	Female	No dementia	High	3	3	3	5	V	Frequent (C3)	no	yes	yes	yes
18	RAD	90+	Female	No dementia	Intermediate	3	2	2	4	III	Moderate (C2)	yes	yes	yes	yes
19	RAD	90+	Female	No dementia	High	2	2	3	3 (A2)	IV	Frequent (C3)	yes	yes	yes	yes
20	RAD	90+	Male	No dementia	High	3	3	3	5 (A3)	V	Frequent (C3)	yes	yes	yes	yes
21	RAD	90+	Male	No dementia	High	2	3	2	3 (A2)	V	Moderate (C2)	yes	yes	yes	yes
22	RAD	90+	Female	No dementia	Intermediate	3	2	3	4 (A3)	III	Frequent (C3)	yes	yes	yes	yes
23	RAD	90+	Male	No dementia	High	3	3	2	5	V	Moderate (C2)	yes	yes	yes	yes
24	ADD	59	Male	Dementia	High	3	3	3	5	VI	Frequent (C3)	yes	yes	yes	yes
25	ADD	61	Male	Dementia	High	3	3	3	5	VI	Frequent (C3)	yes	yes	yes	yes
26	ADD	62	Male	Dementia	High	3	3	3	5 (A3)	VI	Frequent (C3)	no	yes	yes	yes
27	ADD	63	Male	Dementia	High	3	3	3	5	VI	Frequent (C3)	yes	yes	yes	yes
28	ADD	67	Female	Dementia	High	3	3	3	5	VI	Frequent (C3)	yes	yes	yes	yes
29	ADD	76	Male	Dementia	High	3	3	3	5	V	Frequent (C3)	yes	yes	yes	no
30	ADD	77	Male	Dementia	High	2	3	3	3 (A2)	VI	Frequent (C3)	yes	yes	yes	yes
31	ADD	78	Female	Dementia	Intermediate	3	3	2	5	V	Moderate (C2)	yes	yes	yes	no
32	ADD	85	Male	Dementia	High	3	3	3	4	VI	Frequent (C3)	yes	yes	yes	yes
33	ADD	85	Female	Dementia	Intermediate	3	3	2	4	VI	Moderate (C2)	yes	yes	yes	yes
34	ADD	86	Male	Dementia	High	3	3	3	5	V	Frequent (C3)	yes	yes	yes	yes
35	ADD	87	Female	Dementia	High	3	3	3	5	VI	Frequent (C3)	yes	yes	yes	yes
36	ADD	88	Male	Dementia	High	3	3	3	5	VI	Frequent (C3)	yes	yes	no	yes
37	ADD	89	Female	Dementia	Intermediate	3	3	2	5	VI	Moderate (C2)	yes	yes	yes	yes
38	ADD	90+	Female	Dementia	High	3	3	3	5	V	Frequent (C3)	yes	yes	no	yes
39	ADD	90+	Female	Dementia	High	3	3	3	5	VI	Frequent (C3)	yes	yes	yes	yes
40	ADD	90+	Male	Dementia	Intermediate	3	3	2	4	V	Moderate (C2)	yes	yes	yes	yes
41	ADD	90+	Female	Dementia	High	3	3	3	4	VI	Frequent (C3)	yes	yes	yes	yes
42	ADD	90+	Male	Dementia	High	3	3	3	5 (A3)	VI	Frequent (C3)	yes	yes	yes	yes
43	ADD	90+	Male	Dementia	High	3	3	3	4	V	Frequent (C3)	no	yes	yes	yes
Ratio			1F : 1.15M		11 Not: 9 I: 23 H										
Mean		85													
SD		10													

Supplementary Figures

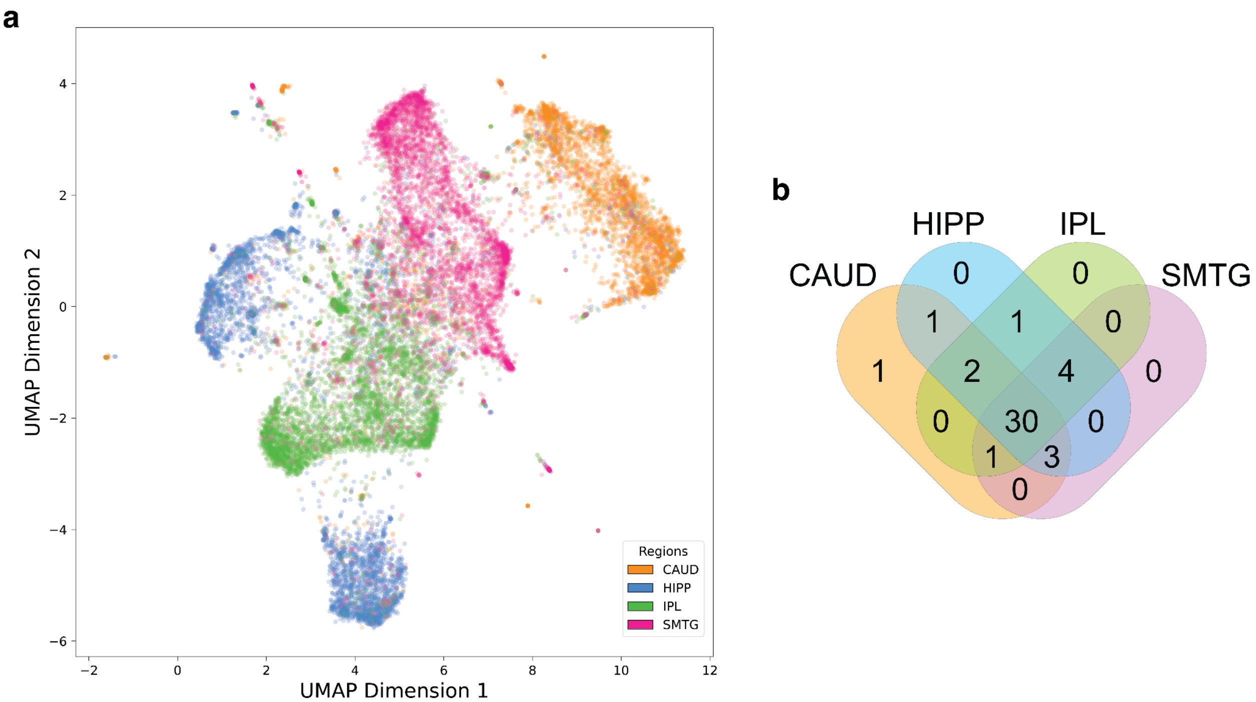

Figure S1. Additional proteomic features. **(a)** UMAP plot of detected protein expressions across four brain regions from the 43 individuals. **(b)** Venn diagram of available individuals among four brain regions. 30 out of 43 individuals had proteomic data across four brain regions.

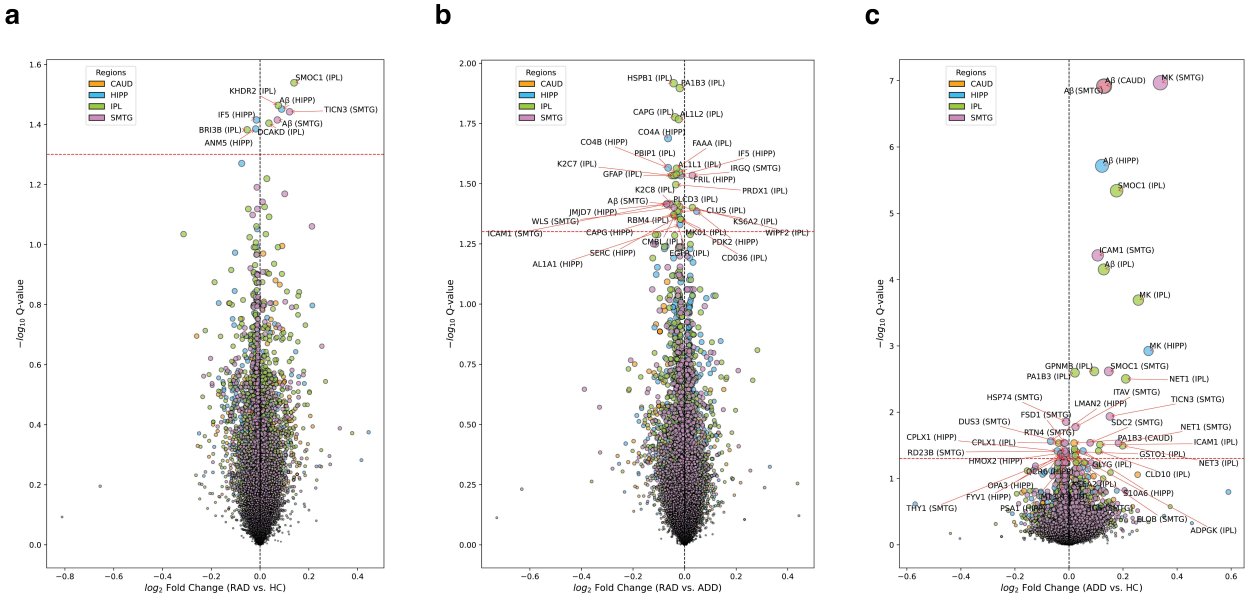

Figure S2. Volcano plot of proteins from four brain regions. **(a)** RAD vs. HC; **(b)** RAD vs. ADD; and **(c)** ADD vs. HC. Two-sided Student's t-test was used to derive the P-values, and Q-values stand for adjusted P-values by Benjamin-Hochberg procedure (FDR=0.05). The dashed red lines are adjusted P-value = 0.05. The circle size stands for -$\log_{10}$(adjusted P-value). Most proteins were differentially expressed between RAD vs. ADD and HC vs. ADD. Most significantly differentially expressed proteins between RAD vs. ADD were lower in the RAD group. Most significantly differentially expressed proteins between HC and ADD were lower in the HC group.

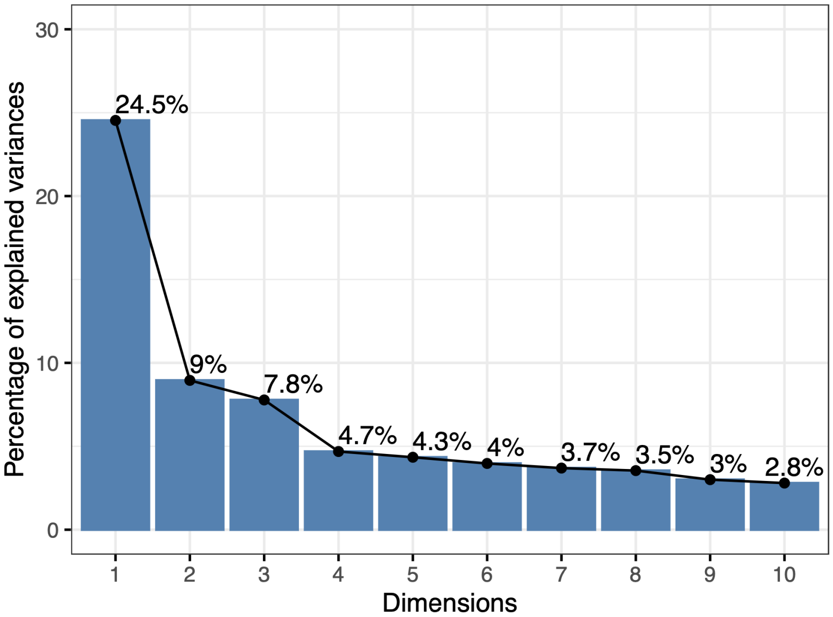

Figure S3. Percentage of explained variances from principal component analysis (PCA). PCA was performed with 33 RAD DEPs in all 4 brain regions (original dimension: 33 × 4 = 132).

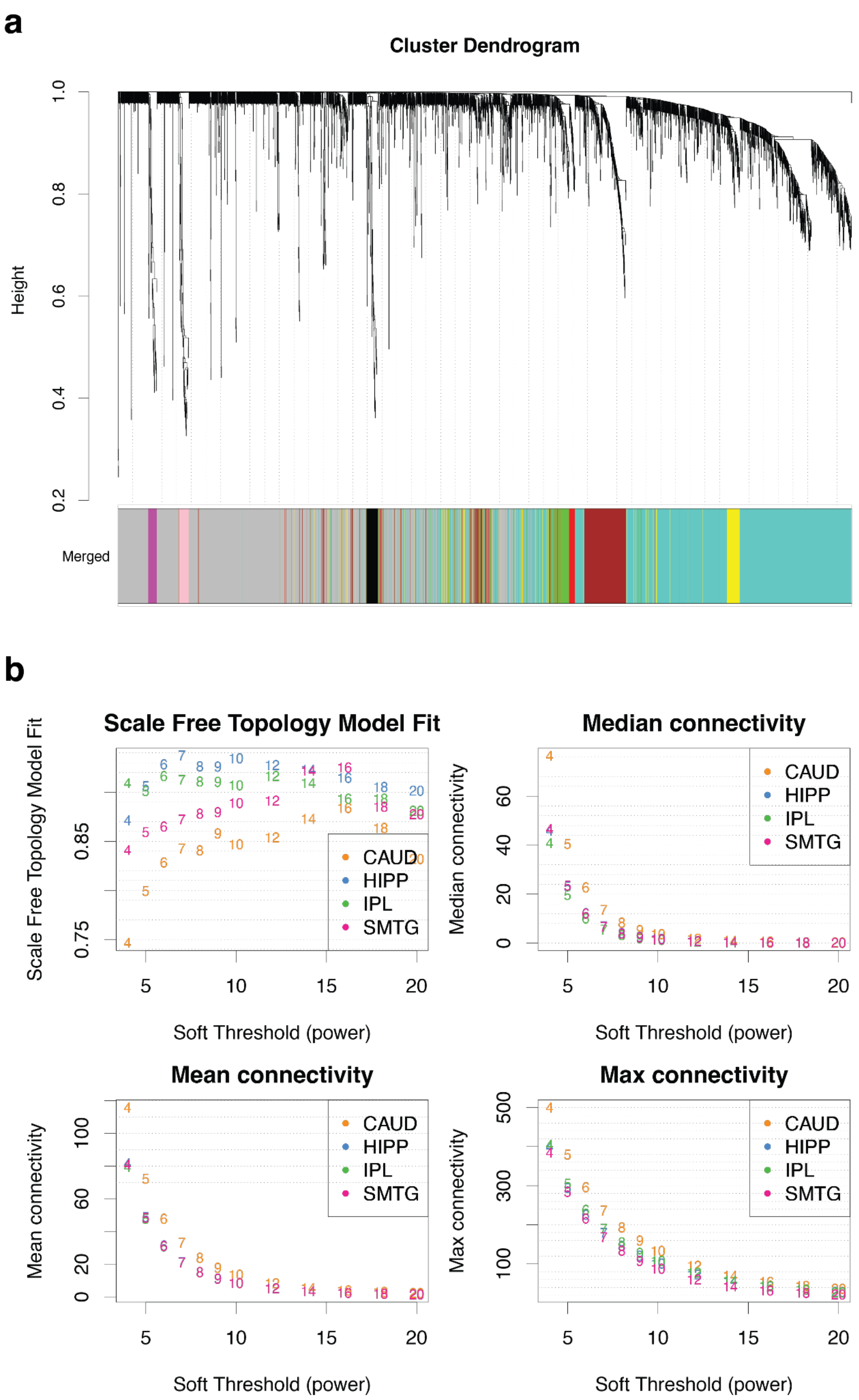

Figure S4. Additional results from the WGCNA co-expression network analysis. **(a)** WGCNA co-expression network cluster dendrogram. **(b)** WGCNA soft threshold vs. scale-free topology, median, mean, and max connectivity.

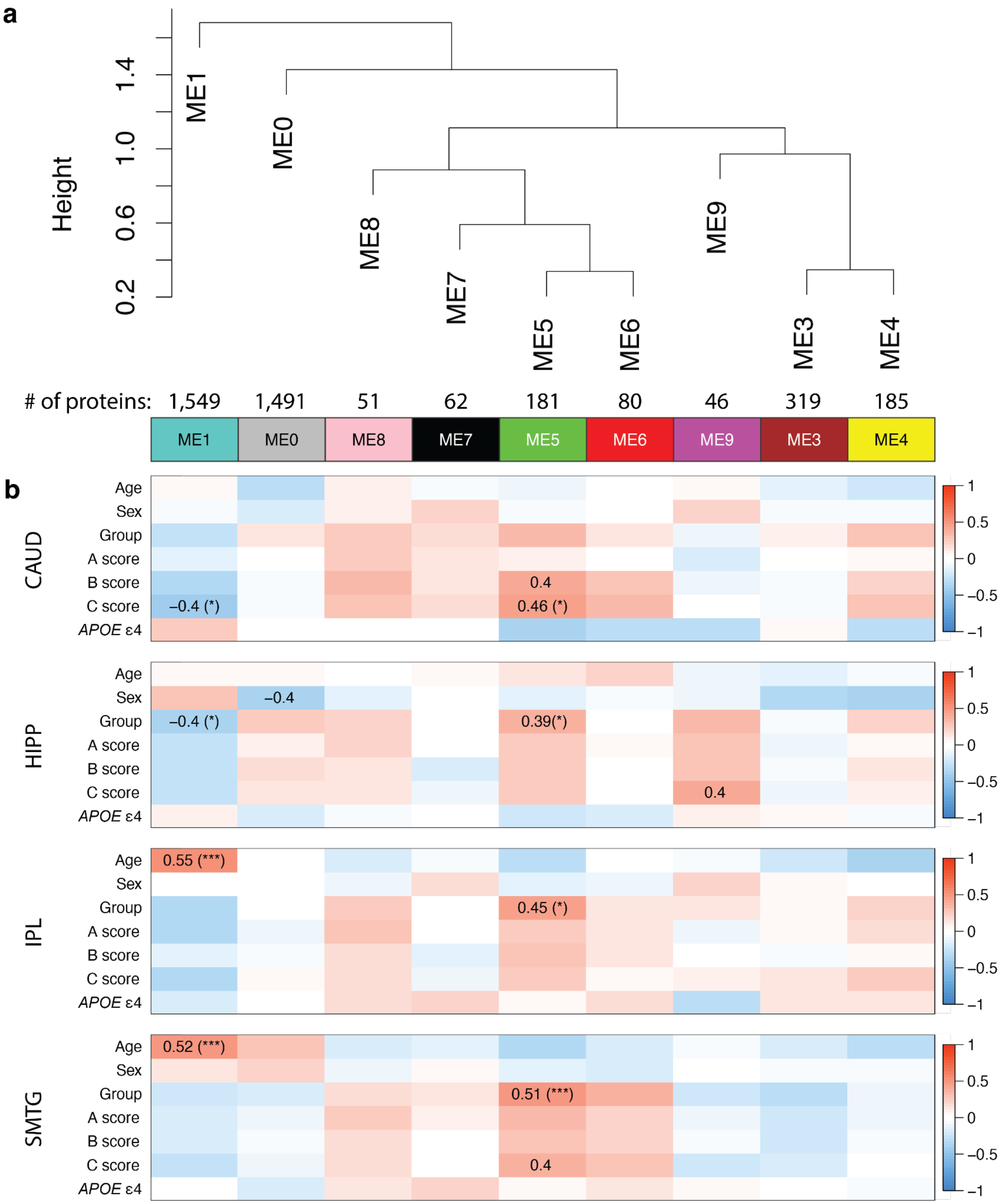

Figure S5. Consensus protein co-expression analysis results with important markers. **(a)** Consensus protein co-expression analysis developed 9 modules across four brain regions. **(b)** Pearson correlation with two-sided P-values was used to evaluate the relationships between different features and eigenprotein expression (threshold = ±0.4 was used to display correlation values if the P-value is not significant). All P-values were adjusted by the Benjamini-Hochberg procedure (FDR cut-off = 0.05). Annotations: * Adjusted P-value < 0.05; ** Adjusted P-value < 0.01, *** Adjusted P-value < 0.001. Source data are provided as a Source Data file.

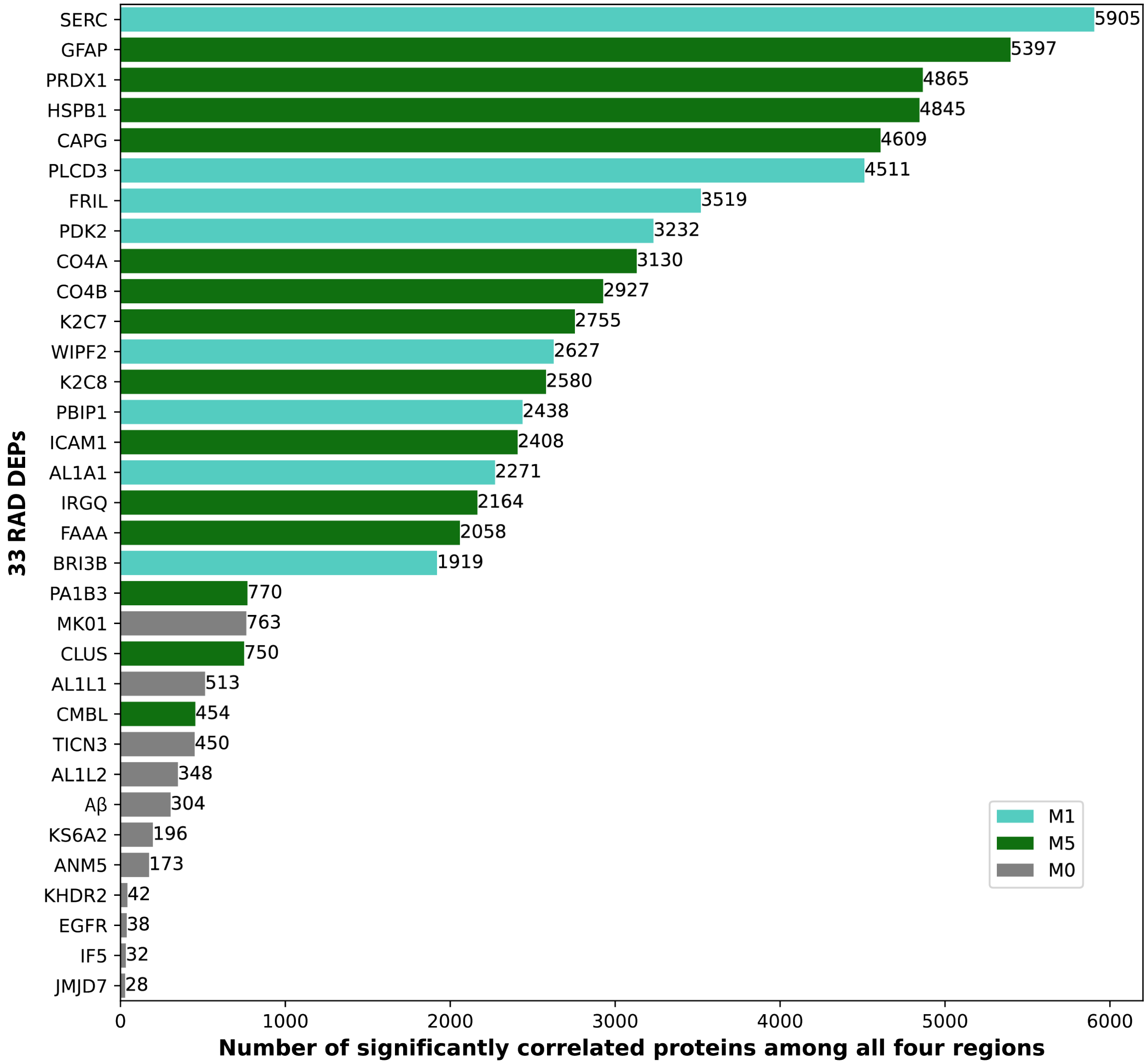

Figure S6. The 33 RAD DEPs were sorted in descending order based on the number of co-expressed proteins. Significance was determined by the P-values < 0.05 from the two-sided Spearman correlation tests. Proteins that were not commonly co-expressed with others were more likely to be attributed to M0 (unassigned module).

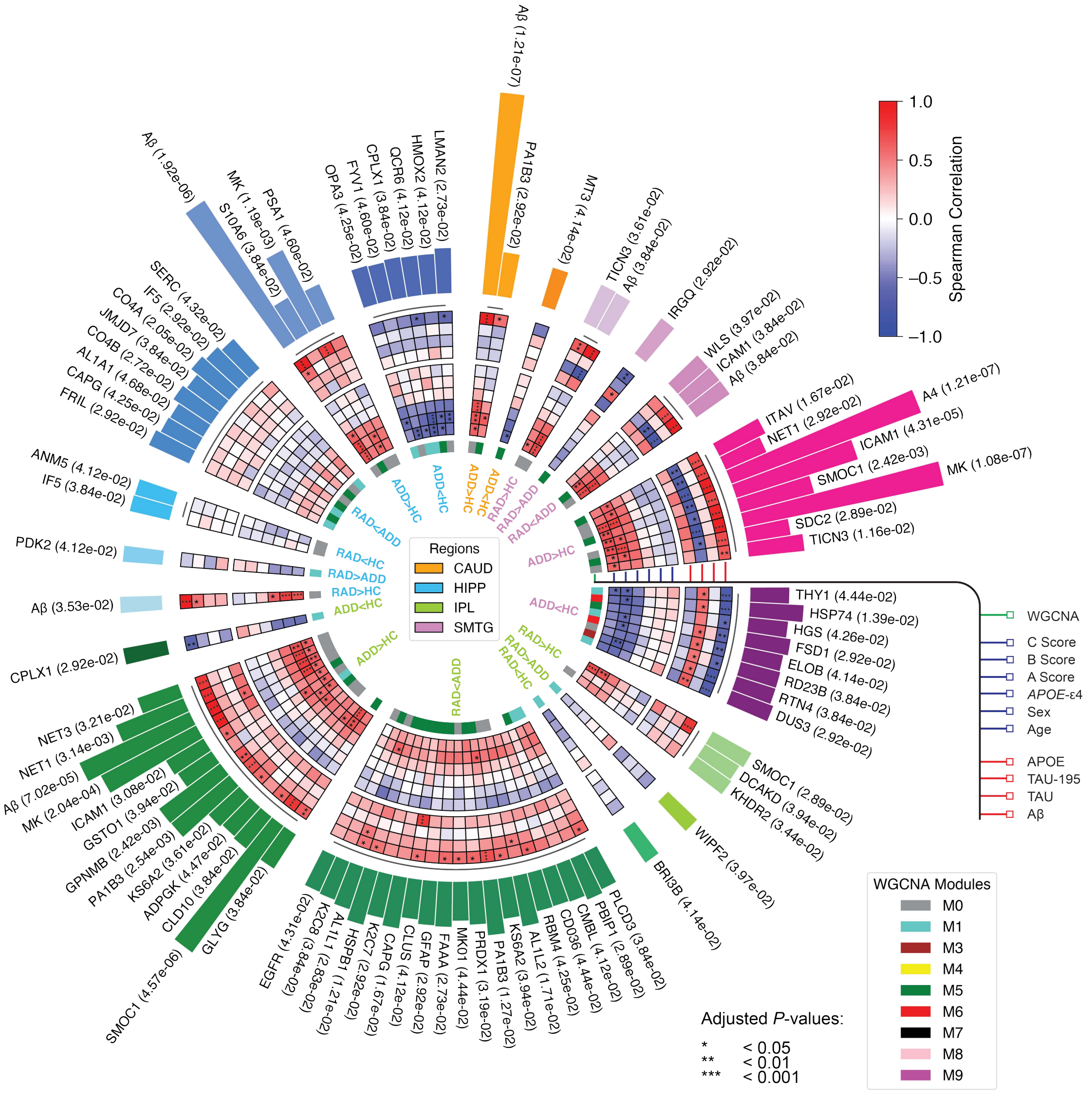

Figure S7. 85 differentially expressed proteins in at least one of the four brain regions. Comparisons include RAD vs. HC, RAD vs. ADD, and ADD vs. HC. Circles from outer rings to inner rings: (i) two-sided Student's t-test adjusted P-value; (ii) spearman correlations with two-sided P-values for four hallmark proteins; (iii) spearman correlations with two-sided P-values with individual features; and (iv) associated WGCNA modules. All P-values were adjusted using the Benjamin-Hochberg procedure (FDR=0.05). Annotations: * Adjusted P-value < 0.05; ** Adjusted P-value < 0.01, *** Adjusted P-value < 0.001.

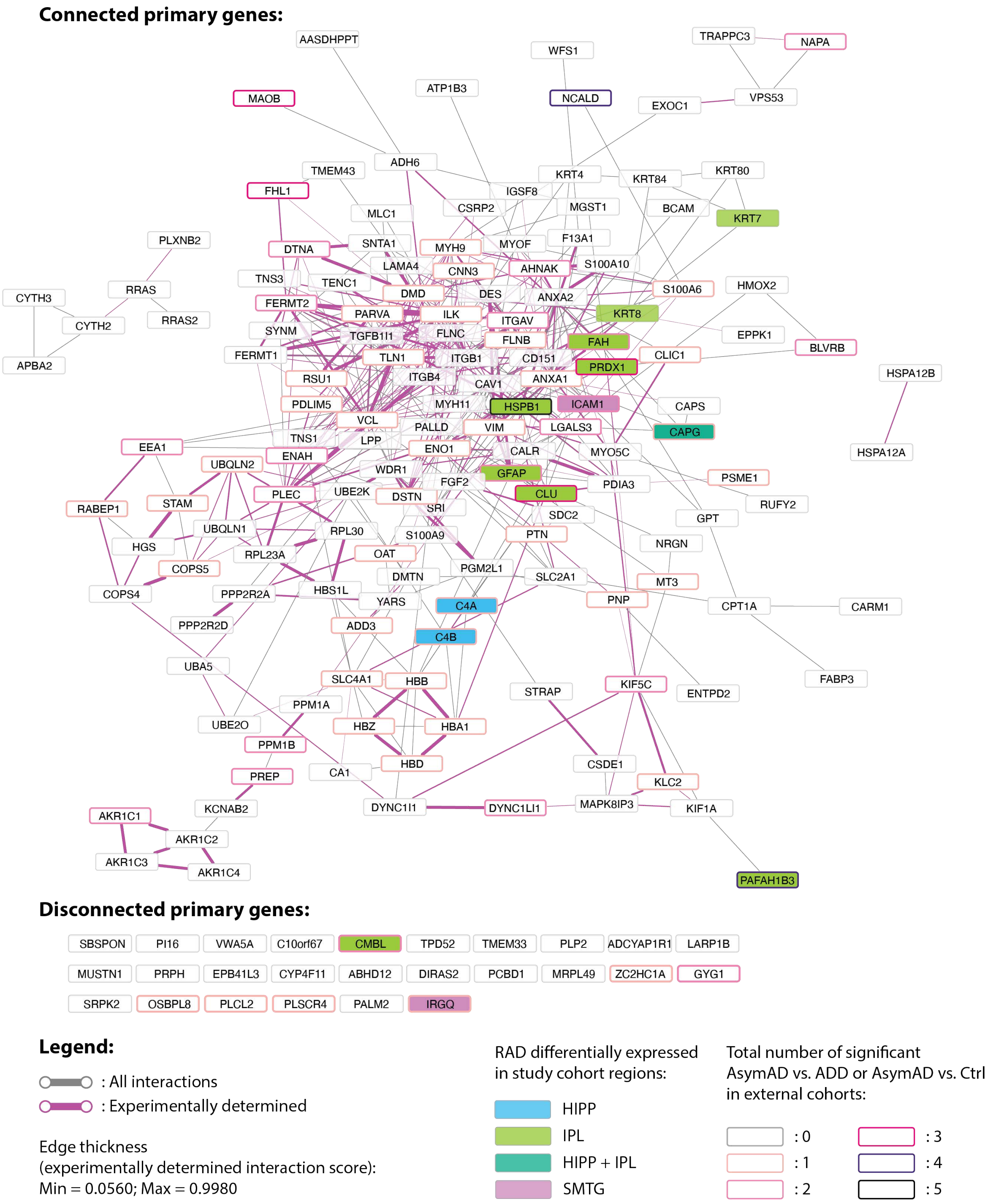

Figure S8. Protein-protein interactions of module 5. Minimum required interaction combined score = 0.4 (default). Magenta-colored edges represent experimentally determined interactions, and the thicknesses of edges indicate the experimentally determined interaction score (Min=0.056; Max=0.998). 14 RAD D EPs were highlighted and colored by brain region. Significant differences between AsymAD vs. ADD or AsymAD vs. Ctrl in external datasets were emphasized by thicker borders. Text in boxes indicates primary genes.

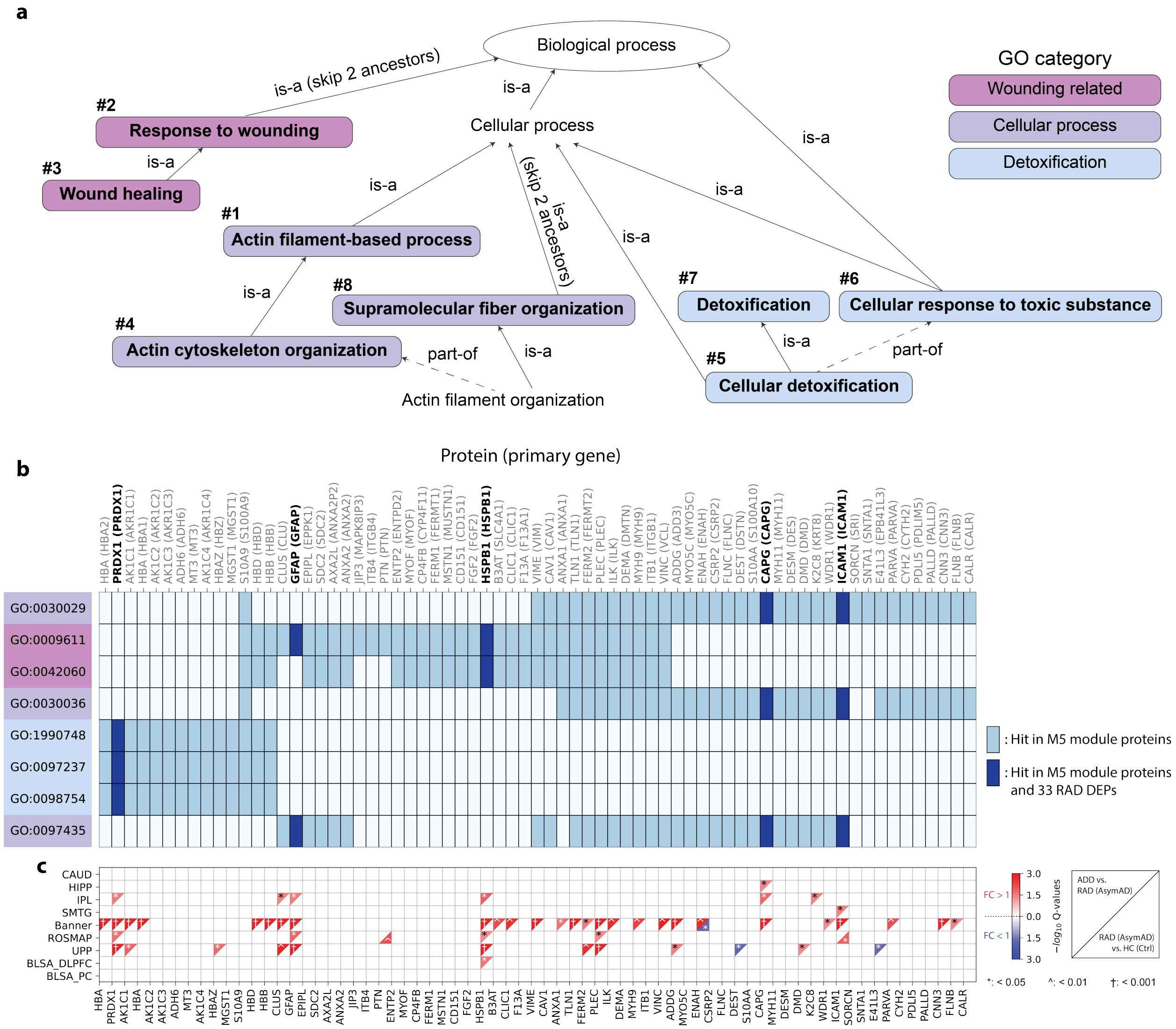

Figure S9. Top enriched GO biological processes in module 5. **(a)** Ancestor chart of top enriched GO biological processes in module 5. **(b)** Top 8 enriched GO biological processes with proteins hit in the M5 module. **(c)** Corresponding differential expression analysis results on ADD vs. RAD (or AsymAD) and RAD (or AsymAD) vs. HC (or Ctrl). All P-values were derived from two-sided Student's t-tests followed by the Benjamin-Hochberg procedure (FDR=0.05). Annotations: * Adjusted P-value < 0.05; ^ Adjusted P-value < 0.01, † Adjusted P-value < 0.001.

Rank	GO ID	GO Term	Z-score: CAUD		HIPP		IPL		SMTG	
			RAD vs HC	RAD vs ADD	RAD vs HC	RAD vs ADD	RAD vs HC	RAD vs ADD	RAD vs HC	RAD vs ADD
1	GO:0030029	Actin filament-based process	2.67	−3.00	−3.67	−4.33	−3.33	−4.67	1.67	−3.67
2	GO:0009611	Response to wounding	2.40	−2.06	−3.94	−4.63	−3.26	−4.97	2.74	−5.14
3	GO:0042060	Wound healing	2.41	−1.67	−3.34	−4.46	−3.71	−4.83	3.16	−5.01
4	GO:0030036	Actin cytoskeleton organization	2.47	−2.47	−3.54	−3.89	−2.83	−4.24	1.77	−3.18
5	GO:1990748	Cellular detoxification	0.28	−0.83	−2.50	−3.05	−0.28	−3.05	0.28	−3.05
6	GO:0097237	Cellular response to toxic substance	0.28	−0.83	−2.50	−3.05	−0.28	−3.05	0.28	−3.05
7	GO:0098754	Detoxification	0.28	−0.83	−2.50	−3.05	−0.28	−3.05	0.28	−3.05
8	GO:0097435	Supramolecular fiber organization	3.65	−2.56	−3.29	−4.38	−2.92	−4.75	2.92	−3.65

GO category: Wounding related (ranks 2, 3); Cellular process (ranks 1, 4, 8); Detoxification (ranks 5, 6, 7)

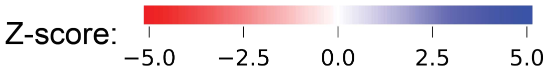

Figure S10. Top 3 enriched GO biological process categories in M5 and their z-scores in study cohorts. Z-scores were calculated based on the number of up/down regulated proteins from the hit count in the query, providing an overall pattern between the RAD groups and GO terms. A negative z-score means that more than half of the proteins had lower expression in RAD than HC or in RAD than ADD (fold-change < 1). Source data are provided as a Source Data file.

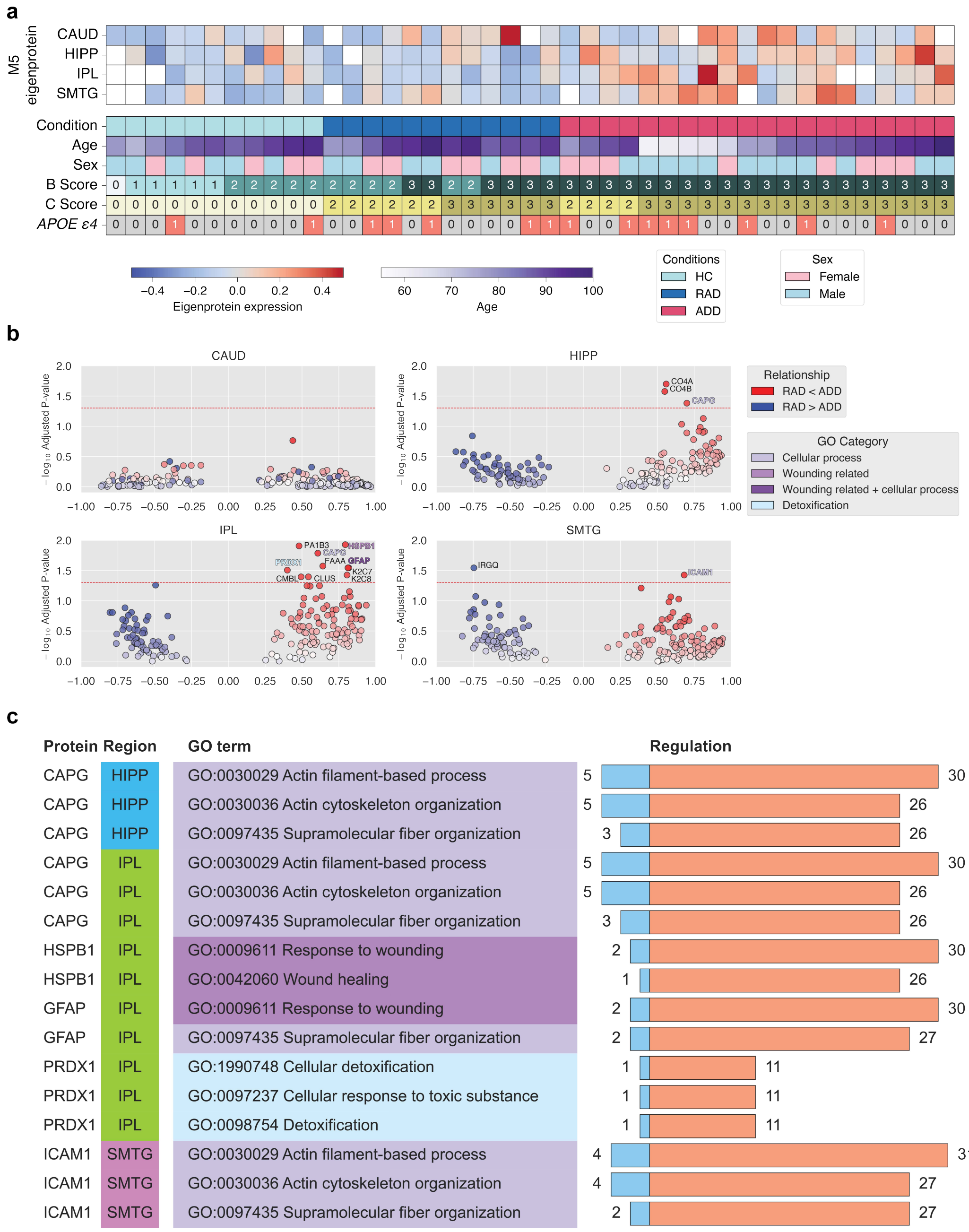

Figure S11. Patterns between protein expressions and M5 eigenprotein expressions. **(a)** Relationships between M5 eigenprotein expressions and clinical factors across four brain regions. Missing values were colored white. Samples are sorted by condition. **(b)** Relationships between M5 eigenprotein and module proteins. X-axis: Pearson correlation coefficient between protein expression and M5 eigenprotein expression. Y-axis: -log10 adjusted P-values from two-sided Student's t-test, followed by the Benjamin-Hochberg procedure (FDR=0.05). Dashed red lines indicate the adjusted P-value = 0.05 threshold. DEPs were colored by GO categories. **(c)** Spearman correlation between 5 DEPs and the overlapping protein expressions among top GO terms. The horizontal bar plot shows the number of positive (orange) and negative (blue) correlations. Protein HBA mapped to two genes HBA1 and HBA2 in GO:1990748, GO:0097237, and GO:0098754.

Rank	GO ID	GO Term	Hit count in query	Hit count in genome	Hit 33 RAD DEPs	Q-value (FDR B&H)
1	GO:0006091	Generation of precursor metabolites and energy	197	594	1 (AL1A1)	4.97E-75
2	GO:1902600	Proton transmembrane transport	137	288	0	1.37E-74
3	GO:0045333	Cellular respiration	116	212	0	1.25E-71
4	GO:0099003	Vesicle-mediated transport in synapse	133	284	0	1.65E-71
5	GO:0006812	Cation transport	314	1458	1 (FRIL)	5.45E-71
6	GO:0007005	Mitochondrion organization	197	629	0	6.33E-71
7	GO:0099536	Synaptic signaling	266	1107	0	9.29E-70
8	GO:0099537	Trans-synaptic signaling	263	1085	0	9.29E-70
9	GO:0098916	Anterograde trans-synaptic signaling	261	1076	0	2.45E-69
10	GO:0007268	Chemical synaptic transmission	261	1076	0	2.45E-69

GO category: Synapse | Energy | Transport | Cellular process

Figure S12. Top 4 enriched GO biological process categories in M1 and their enrichment analysis results. Source data are provided as a Source Data file.

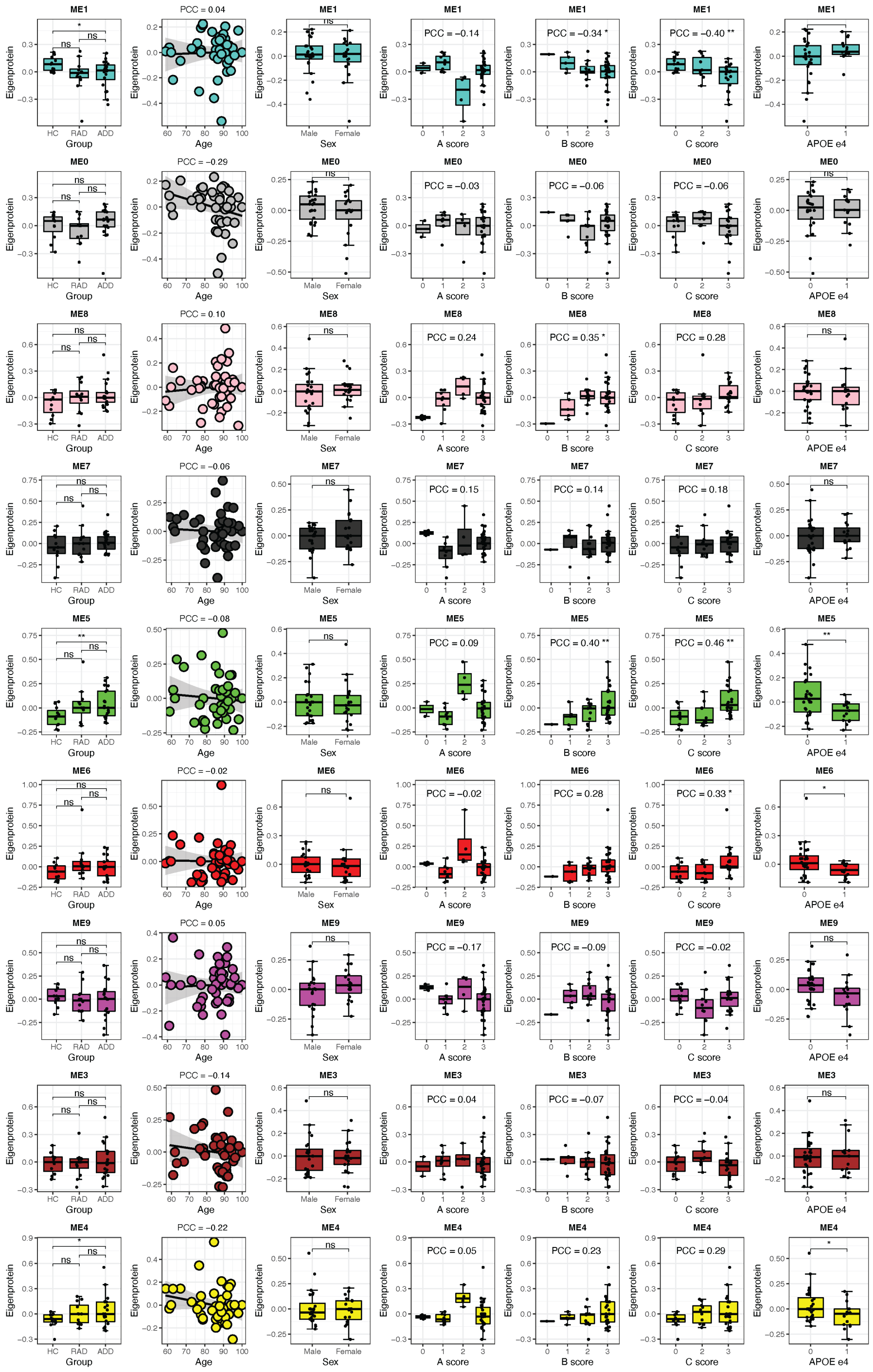

Figure S13. Eigenprotein expressions versus group, age, sex, A score, B score, C score, and *APOE* Ɛ4 allele in CAUD. The number of samples in the study cohort: HC = 11, RES = 12, ADD = 20. All P-values were derived by two-sided Student's t-test, followed by the Benjamin-Hochberg procedure (FDR=0.05). Annotations: * Adjusted P-value < 0.05; ** Adjusted P-value < 0.01, *** Adjusted P-value < 0.001, **** Adjusted P-value < 0.0001. For the boxplots, the interior horizontal line represents the median value, the upper and lower box edges represent the 75th and 25th percentile, and the upper and lower bars represent the 90th and 10th percentiles, respectively. Source data are provided as a Source Data file.

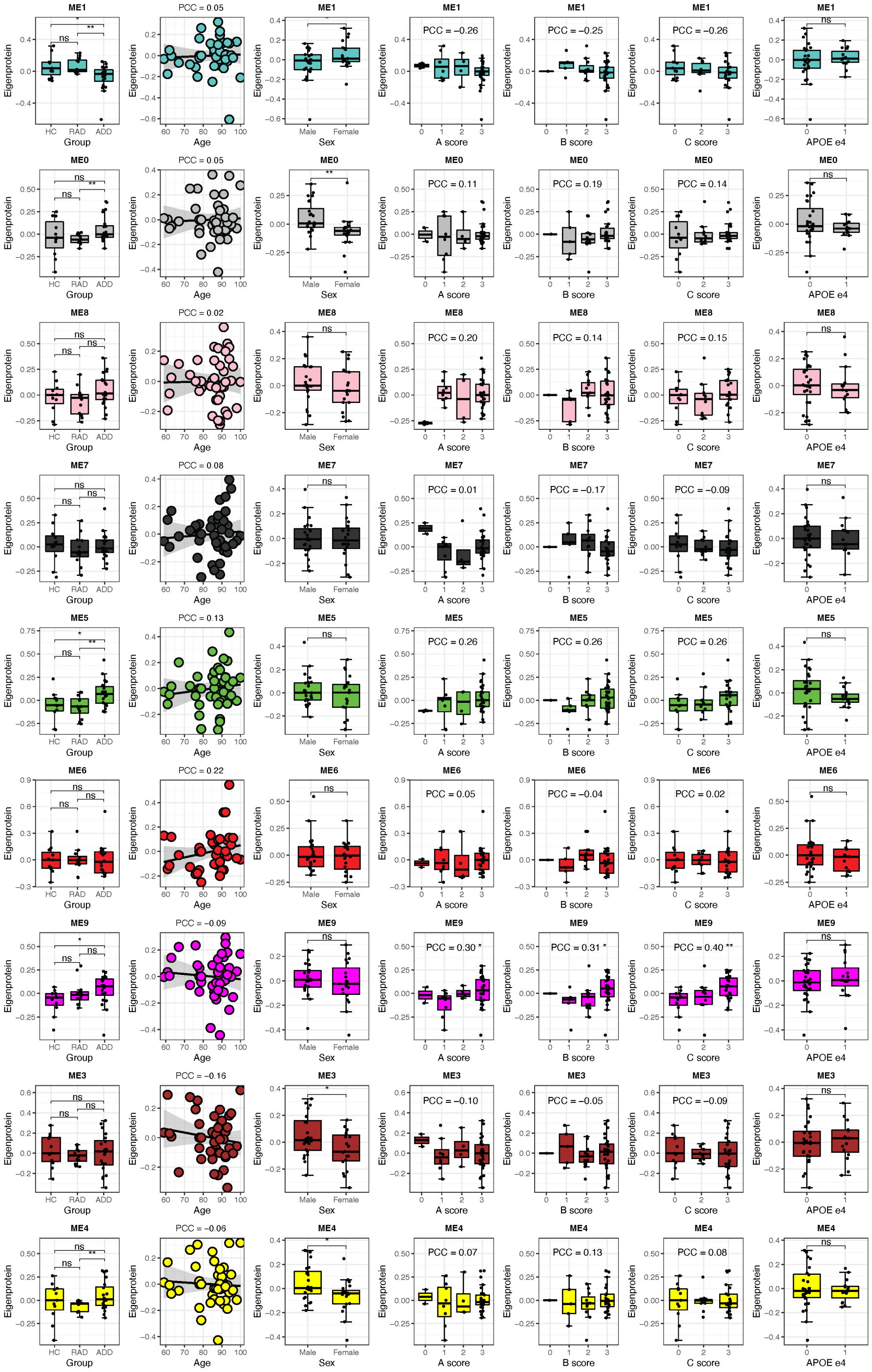

Figure S14. Eigenprotein expressions versus group, age, sex, A score, B score, C score, and *APOE* Ɛ4 allele in HIPP. The number of samples in the study cohort: HC = 11, RES = 12, ADD = 20. All P-values were derived by two-sided Student's t-test, followed by the Benjamin-Hochberg procedure (FDR=0.05). Annotations: * Adjusted P-value < 0.05; ** Adjusted P-value < 0.01, *** Adjusted P-value < 0.001, **** Adjusted P-value < 0.0001. For the boxplots, the interior horizontal line represents the median value, the upper and lower box edges represent the 75th and 25th percentile, and the upper and lower bars represent the 90th and 10th percentiles, respectively. Source data are provided as a Source Data file.

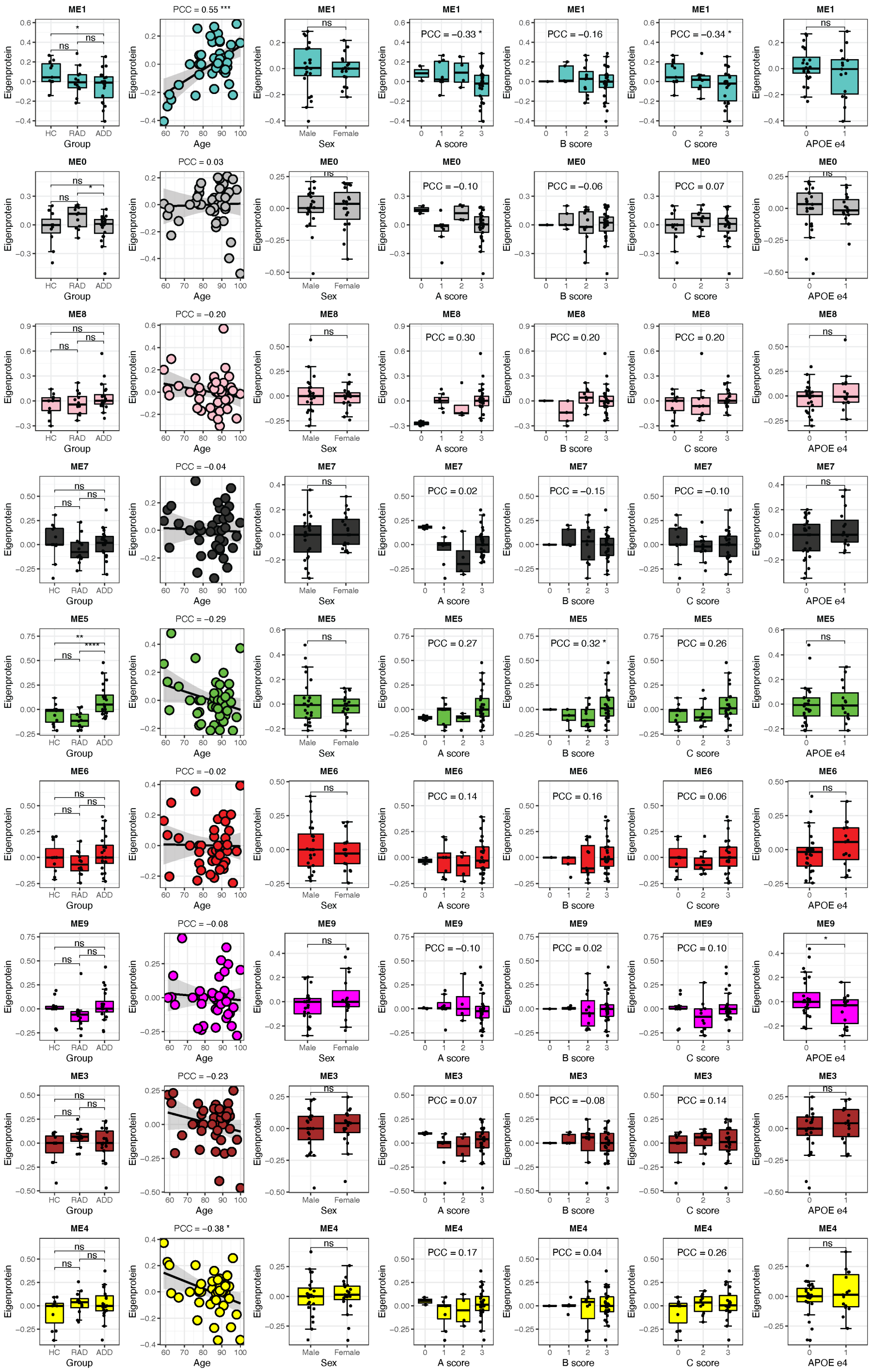

Figure S15. Eigenprotein expressions versus group, age, sex, A score, B score, C score, and *APOE* Ɛ4 allele in IPL. The number of samples in the study cohort: HC = 11, RES = 12, ADD = 20. All P-values were derived by two-sided Student's t-test, followed by the Benjamin-Hochberg procedure (FDR=0.05). Annotations: * Adjusted P-value < 0.05; ** Adjusted P-value < 0.01, *** Adjusted P-value < 0.001, **** Adjusted P-value < 0.0001. For the boxplots, the interior horizontal line represents the median value, the upper and lower box edges represent the 75th and 25th percentile, and the upper and lower bars represent the 90th and 10th percentiles, respectively. Source data are provided as a Source Data file.

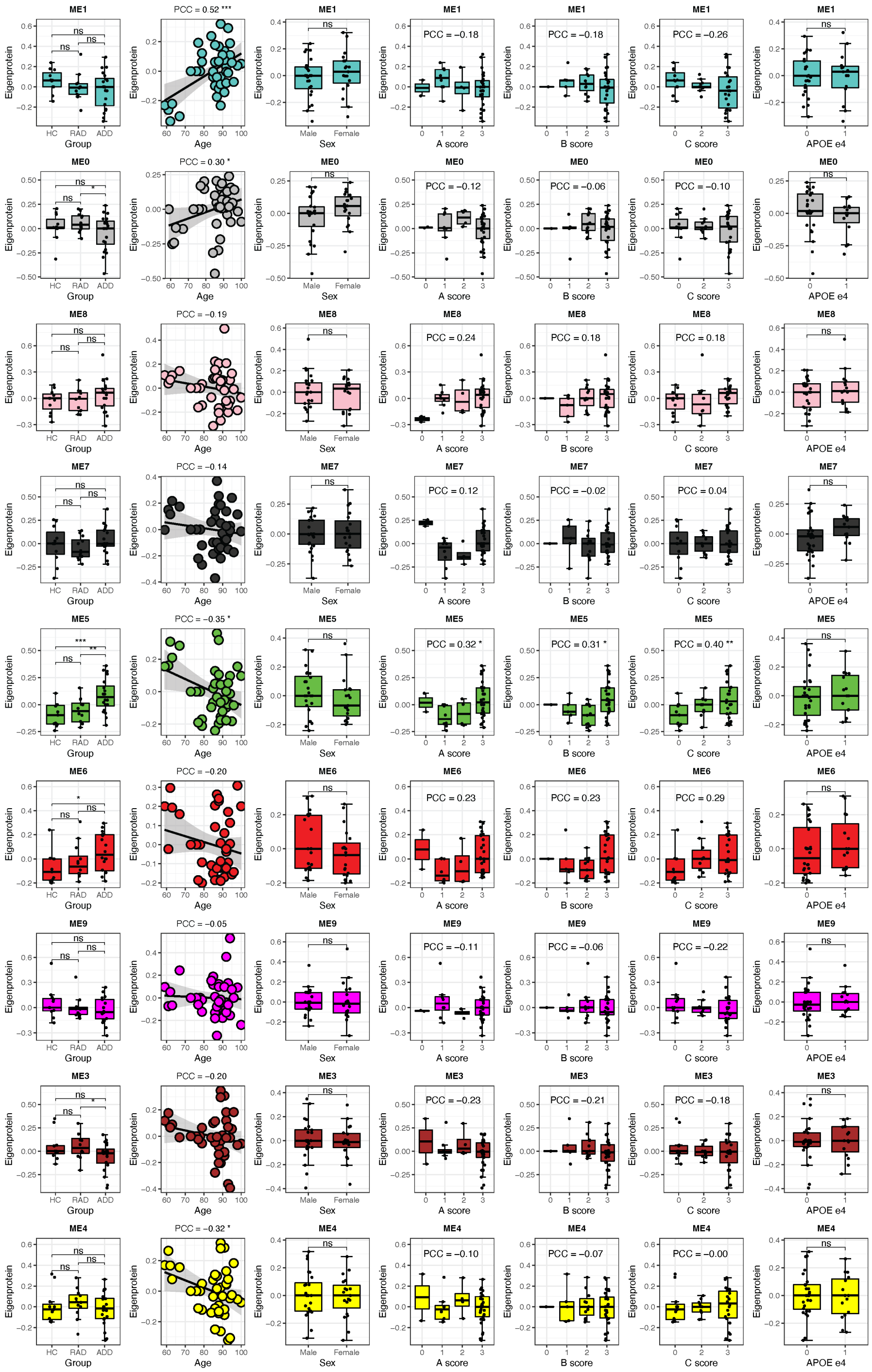

Figure S16. Eigenprotein expressions versus group, age, sex, A score, B score, C score, and *APOE* Ɛ4 allele in SMTG. The number of samples in the study cohort: HC = 11, RES = 12, ADD = 20. All P-values were derived by two-sided Student's t-test, followed by the Benjamin-Hochberg procedure (FDR=0.05). Annotations: * Adjusted P-value < 0.05; ** Adjusted P-value < 0.01, *** Adjusted P-value < 0.001, **** Adjusted P-value < 0.0001. For the boxplots, the interior horizontal line represents the median value, the upper and lower box edges represent the 75th and 25th percentile, and the upper and lower bars represent the 90th and 10th percentiles, respectively. Source data are provided as a Source Data file.

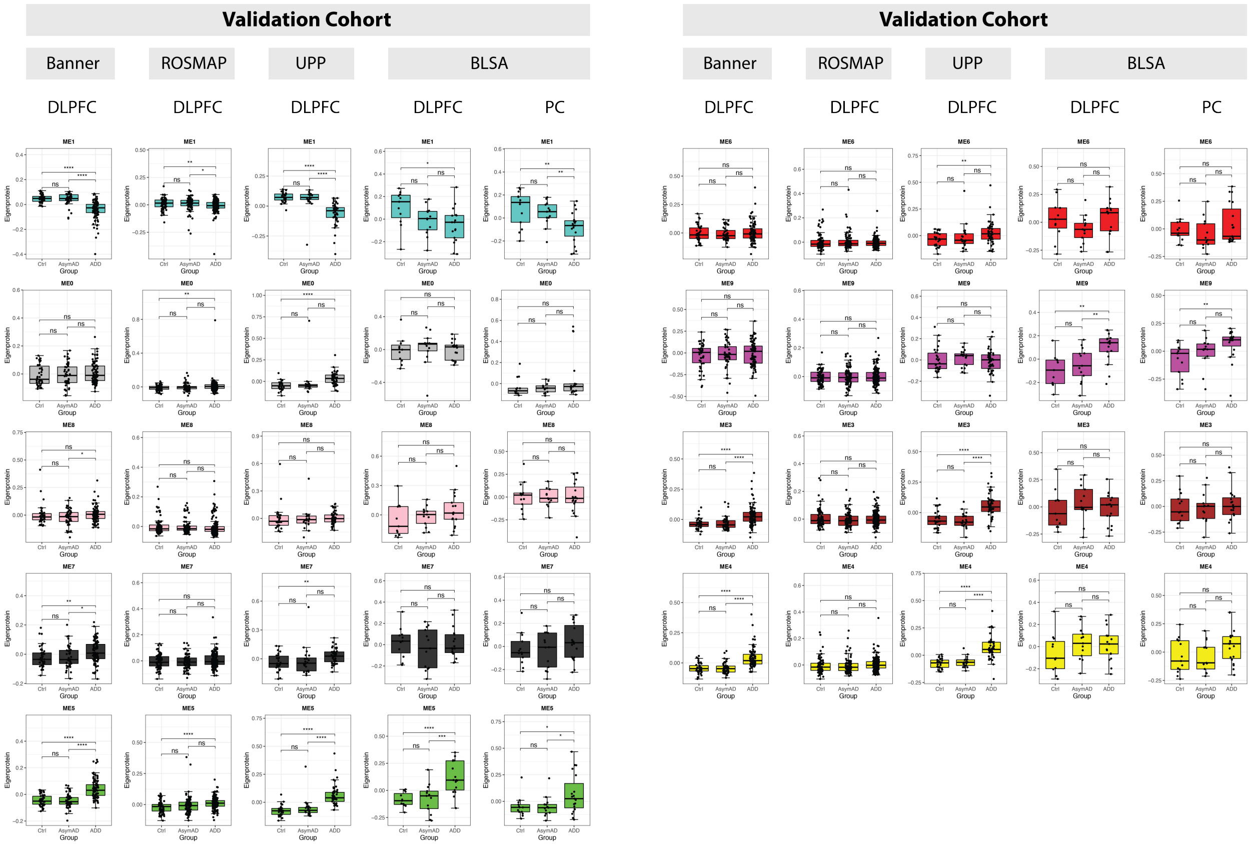

Figure S17. Nine co-expression modules from the study cohort were validated by external datasets. AsymAD is a mix of preclinical AD and RAD cases. Ctrl includes HC. Banner stands for Banner Sun Health Research Institute dataset; ROSMAP for Religious Orders Study and Rush Memory and Aging Project dataset; UPP for UPenn Proteomics study dataset; and BLSA for Baltimore Longitudinal Study of Aging dataset. Abbreviateions: dorsolateral prefrontal cortex (DLPFC), precuneus (PC). The number of samples in the external validation cohorts: Banner (Ctrl = 42, AsymAD = 45, ADD = 92), ROSMAP (Ctrl = 78, AsymAD = 89, ADD = 162), UPP (Ctrl = 26, AsymAD = 20, ADD = 49), and BLSA (DLPFC: Ctrl = 11, AsymAD = 13, ADD = 17; PC: Ctrl = 13, AsymAD = 13, ADD = 19). All P-values were derived by two-sided Student's t-test, followed by the Benjamin-Hochberg procedure (FDR=0.05). Annotations: * Adjusted P-value < 0.05; ** Adjusted P-value < 0.01, *** Adjusted P-value < 0.001, **** Adjusted P-value < 0.0001. For the boxplots, the interior horizontal line represents the median value, the upper and lower box edges represent the 75th and 25th percentile, and the upper and lower bars represent the 90th and 10th percentiles, respectively. Source data are provided as a Source Data file.

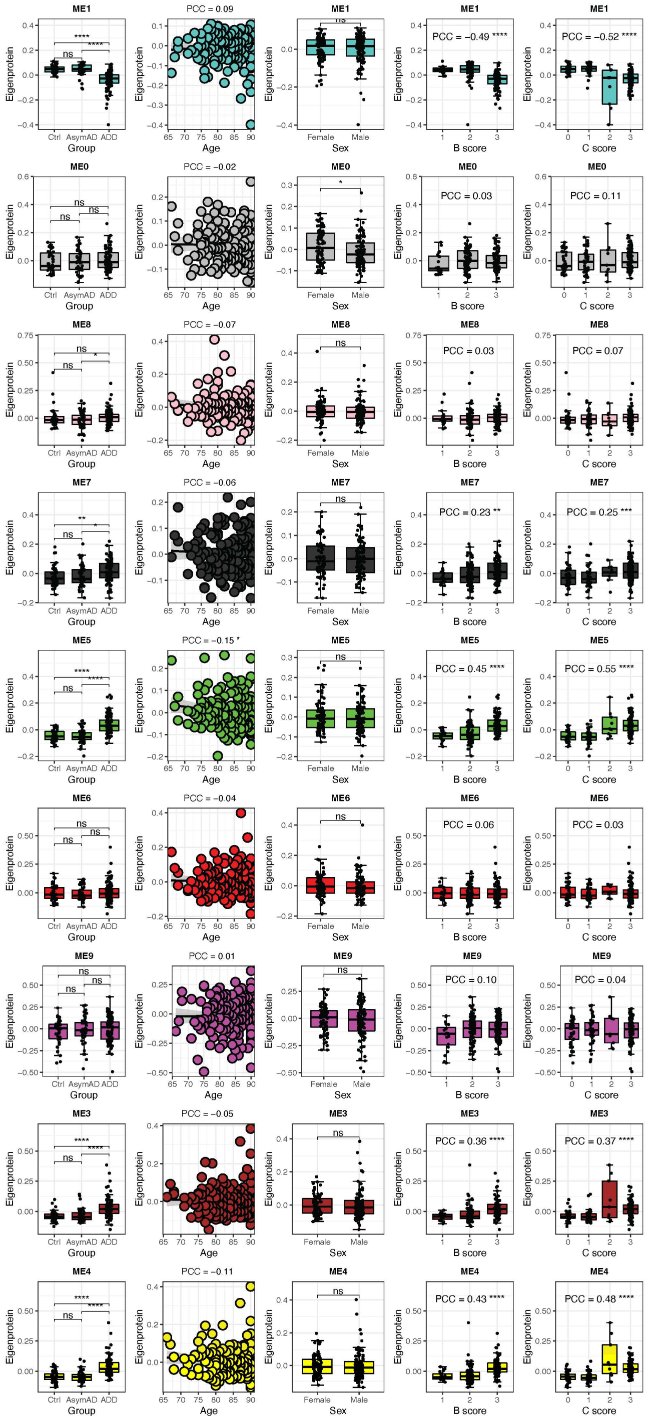

Figure S18. Eigenprotein expressions versus group, age, sex, B score, and C score in Banner Sun Health Research Institute (Banner) dataset dorsolateral prefrontal cortex (DLPFC). The number of samples in the Banner dataset: Ctrl = 42, AsymAD = 45, ADD = 92. All P-values were derived by two-sided Student's t-test, followed by the Benjamin-Hochberg procedure (FDR=0.05). Annotations: * Adjusted P-value < 0.05; ** Adjusted P-value < 0.01, *** Adjusted P-value < 0.001, **** Adjusted P-value < 0.0001. For the boxplots, the interior horizontal line represents the median value, the upper and lower box edges represent the 75th and 25th percentile, and the upper and lower bars represent the 90th and 10th percentiles, respectively. Source data are provided as a Source Data file.

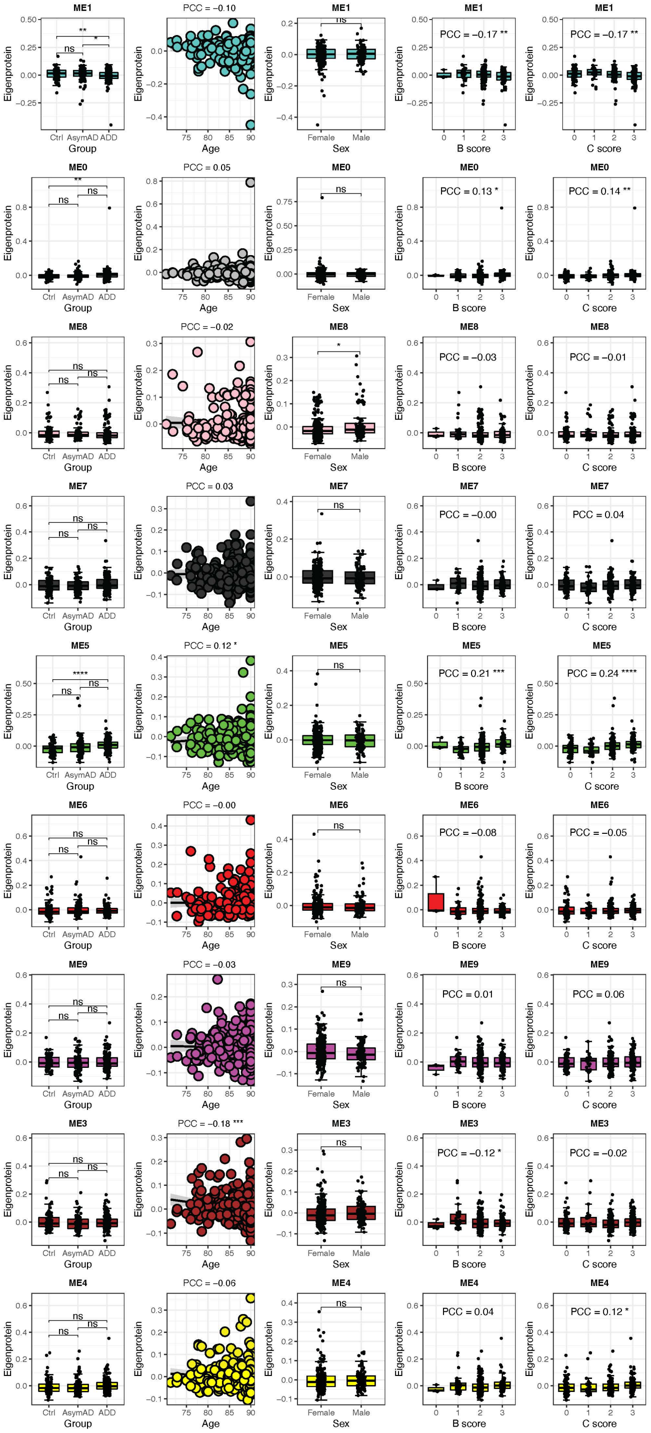

Figure S19. Eigenprotein expressions versus group, age, sex, B score, and C score in Religious Orders Study and Rush Memory and Aging Project (ROSMAP) dataset dorsolateral prefrontal cortex (DLPFC). The number of samples in the ROSMAP dataset: Ctrl = 78, AsymAD = 89, ADD = 162. All P-values were derived by two-sided Student's t-test, followed by the Benjamin-Hochberg procedure (FDR=0.05). Annotations: * Adjusted P-value < 0.05; ** Adjusted P-value < 0.01, *** Adjusted P-value < 0.001, **** Adjusted P-value < 0.0001. For the boxplots, the interior horizontal line represents the median value, the upper and lower box edges represent the 75th and 25th percentile, and the upper and lower bars represent the 90th and 10th percentiles, respectively. Source data are provided as a Source Data file.

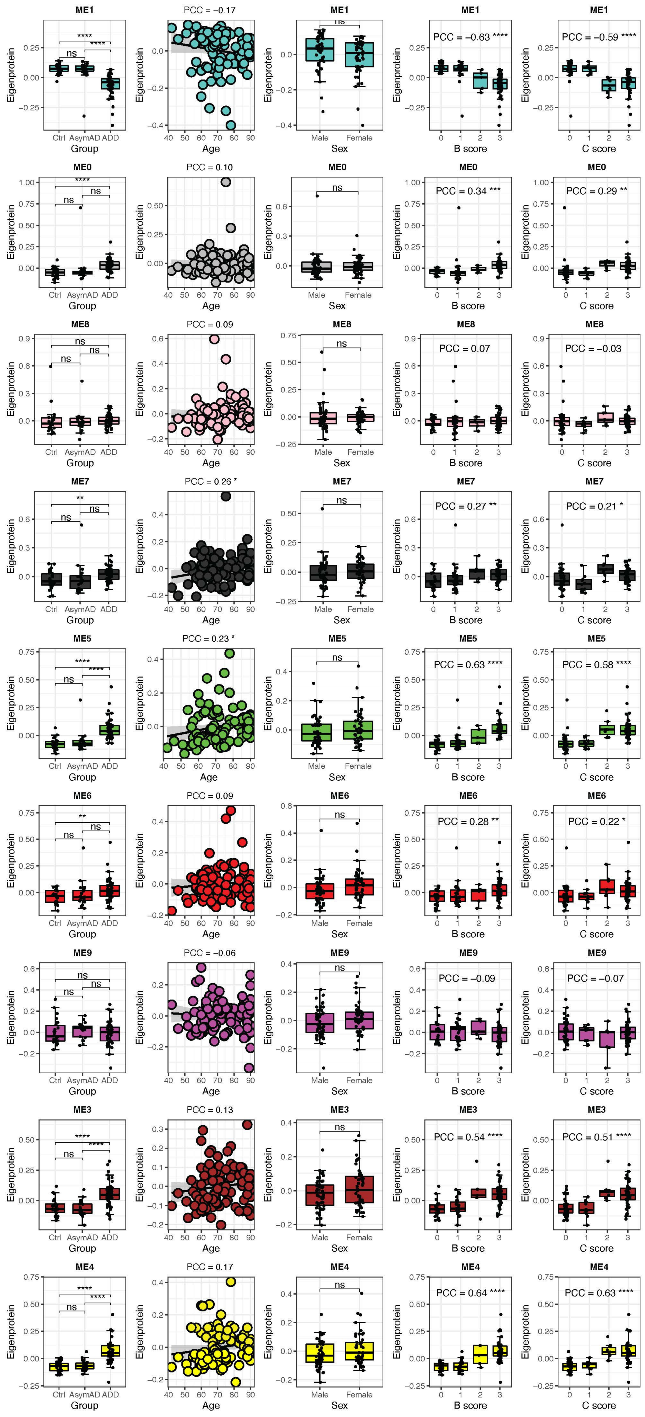

Figure S20. Eigenprotein expressions versus group, age, sex, B score, and C score in UPenn Proteomics study (UPP) dataset dorsolateral prefrontal cortex (DLPFC). The number of samples in the UPP dataset: Ctrl = 26, AsymAD = 20, ADD = 49. All P-values were derived by two-sided Student's t-test, followed by the Benjamin-Hochberg procedure (FDR=0.05). Annotations: * Adjusted P-value < 0.05; ** Adjusted P-value < 0.01, *** Adjusted P-value < 0.001, **** Adjusted P-value < 0.0001. For the boxplots, the interior horizontal line represents the median value, the upper and lower box edges represent the 75th and 25th percentile, and the upper and lower bars represent the 90th and 10th percentiles, respectively. Source data are provided as a Source Data file.

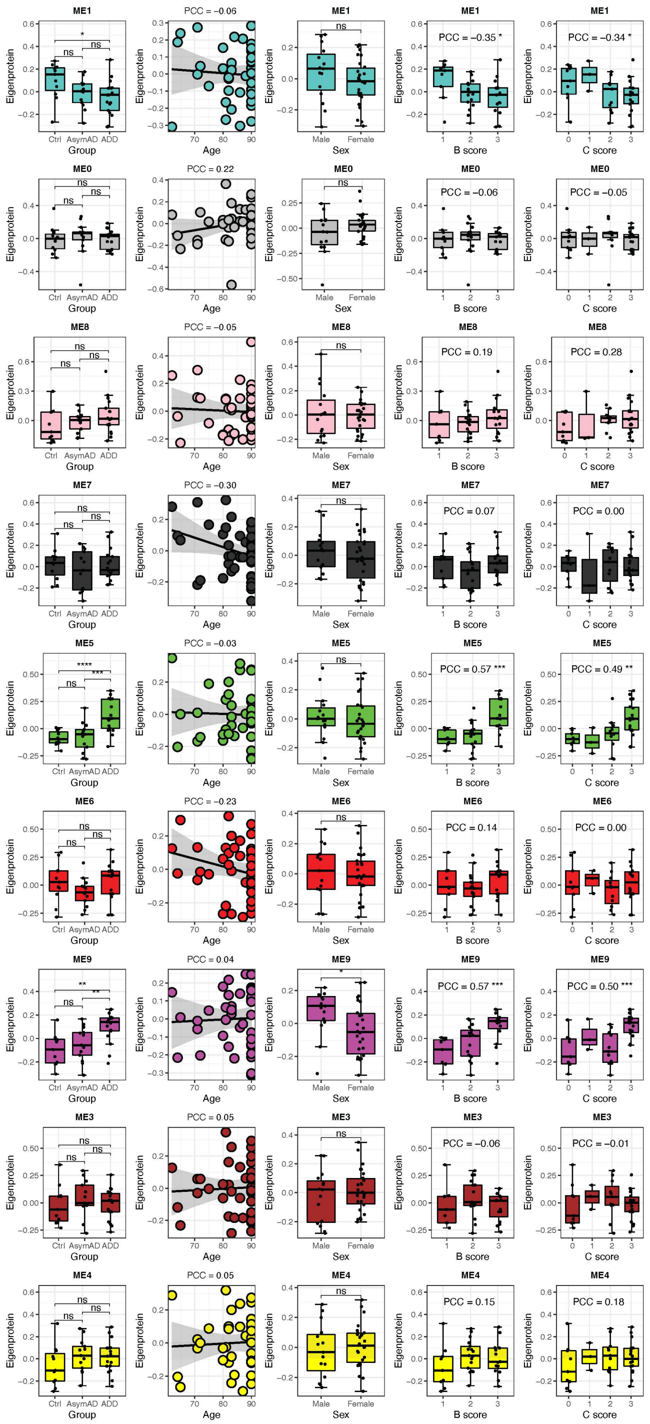

Figure S21. Eigenprotein expressions versus group, age, sex, B score, and C score in Baltimore Longitudinal Study of Aging (BLSA) dataset dorsolateral prefrontal cortex (DLPFC). The number of samples in the BLSA DLPFC dataset: Ctrl = 11, AsymAD = 13, ADD = 17. All P-values were derived by two-sided Student's t-test, followed by the Benjamin-Hochberg procedure (FDR=0.05). Annotations: * Adjusted P-value < 0.05; ** Adjusted P-value < 0.01, *** Adjusted P-value < 0.001, **** Adjusted P-value < 0.0001. For the boxplots, the interior horizontal line represents the median value, the upper and lower box edges represent the 75th and 25th percentile, and the upper and lower bars represent the 90th and 10th percentiles, respectively. Source data are provided as a Source Data file.

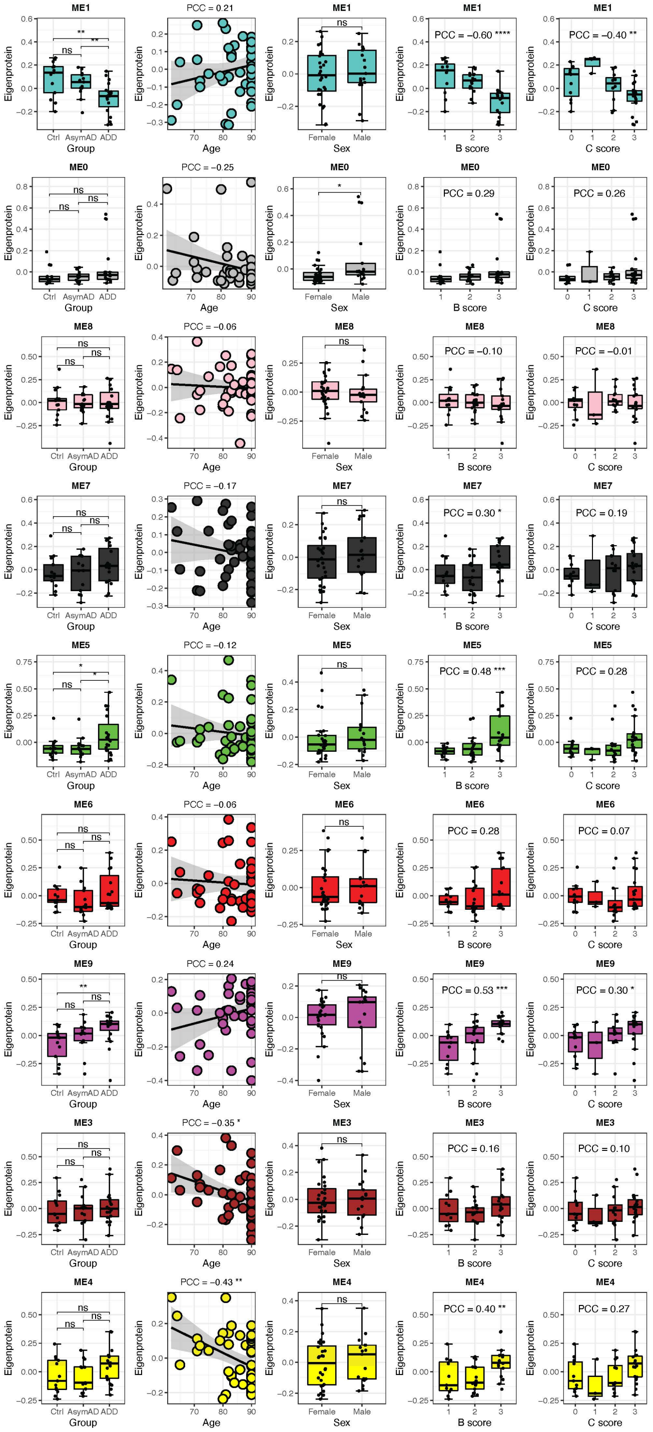

Figure S22. Eigenprotein expressions versus group, age, sex, B score, and C score in Baltimore Longitudinal Study of Aging (BLSA) dataset precuneus (PC) region. The number of samples in the BLSA PC dataset: Ctrl = 13, AsymAD = 13, ADD = 19. All P-values were derived by two-sided Student's t-test, followed by the Benjamin-Hochberg procedure (FDR=0.05). Annotations: * Adjusted P-value < 0.05; ** Adjusted P-value < 0.01, *** Adjusted P-value < 0.001, **** Adjusted P-value < 0.0001. For the boxplots, the interior horizontal line represents the median value, the upper and lower box edges represent the 75th and 25th percentile, and the upper and lower bars represent the 90th and 10th percentiles, respectively. Source data are provided as a Source Data file.

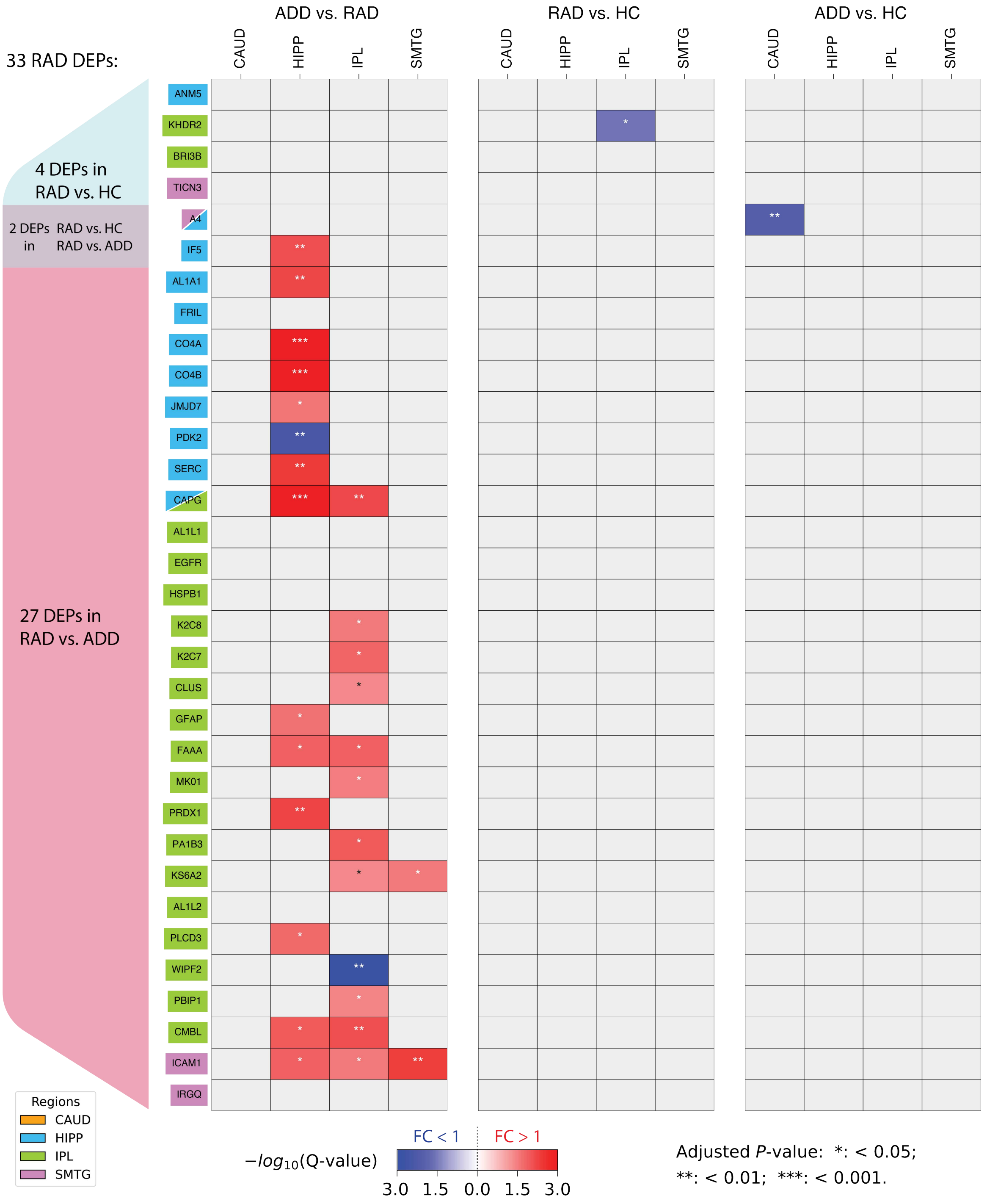

Figure S23. An orthogonal analysis compared 33 DEPs using linear models. Specifically, linear models were used to regress the conditions (HC, RAD, and ADD) by protein expression, with the following control variables: age, sex (female=0, male=1), A score (0-3), B score (0-3), and C score (0-3). Insignificant results are grey; fold change (FC) < 1 was colored in blue, FC > 1 was colored in red; and colors are the same for -log10(Adjusted P-value) ≥ 3). All P-values were adjusted for multiple comparisons by the Benjamin-Hochberg procedure (FDR cut-off = 0.05). Annotations: * Adjusted P-value < 0.05; ** Adjusted P-value < 0.01, *** Adjusted P-value < 0.001. Source data are provided as a Source Data file.
